# Integrating genetics with newborn metabolomics in infantile hypertrophic pyloric stenosis

**DOI:** 10.1007/s11306-020-01763-2

**Published:** 2021-01-08

**Authors:** João Fadista, Line Skotte, Julie Courraud, Frank Geller, Sanne Gørtz, Jan Wohlfahrt, Mads Melbye, Arieh S. Cohen, Bjarke Feenstra

**Affiliations:** 1 Department of Epidemiology Research, Statens Serum Institut, Artillerivej 5, 2300 Copenhagen, Denmark; 2 Department of Clinical Sciences, Lund University Diabetes Centre, Malmö, Sweden; 3 Institute for Molecular Medicine Finland (FIMM), University of Helsinki, Helsinki, Finland; 4 Danish Center for Neonatal Screening, Clinical Mass Spectrometry, Statens Serum Institut, Copenhagen, Denmark; 5 Department of Clinical Medicine, University of Copenhagen, Copenhagen, Denmark; 6 Department of Medicine, Stanford University School of Medicine, Stanford, CA USA

## Abstract

**Introduction:**

Infantile hypertrophic pyloric stenosis (IHPS) is caused by hypertrophy of the pyloric sphincter muscle.

**Objectives:**

Since previous reports have implicated lipid metabolism, we aimed to (1) investigate associations between IHPS and a wide array of lipid-related metabolites in newborns, and (2) address whether detected differences in metabolite levels were likely to be driven by genetic differences between IHPS cases and controls or by differences in early life feeding patterns.

**Methods:**

We used population-based random selection of IHPS cases and controls born in Denmark between 1997 and 2014. We randomly took dried blood spots of newborns from 267 pairs of IHPS cases and controls matched by sex and day of birth. We used a mixed-effects linear regression model to evaluate associations between 148 metabolites and IHPS in a matched case–control design.

**Results:**

The phosphatidylcholine PC(38:4) showed significantly lower levels in IHPS cases (*P* = 4.68 × 10^−8^) as did six other correlated metabolites (four phosphatidylcholines, acylcarnitine AC(2:0), and histidine). Associations were driven by 98 case–control pairs born before 2009, when median age at sampling was 6 days. No association was seen in 169 pairs born in 2009 or later, when median age at sampling was 2 days. More IHPS cases than controls had a diagnosis for neonatal difficulty in feeding at breast (*P* = 6.15 × 10^−3^). Genetic variants known to be associated with PC(38:4) levels did not associate with IHPS.

**Conclusions:**

We detected lower levels of certain metabolites in IHPS, possibly reflecting different feeding patterns in the first days of life.

**Supplementary information:**

The online version of this article (doi:10.1007/s11306-020-01763-2) contains supplementary material, which is available to authorized users.

## Introduction

Infantile hypertrophic pyloric stenosis (IHPS), a disease typically appearing between two and eight weeks after birth, is characterized by hypertrophy of the pyloric sphincter smooth muscle, which leads to obstruction of the gastric outlet (MacMahon 2008). IHPS has a population incidence between 1 and 4 per 1000 live births (Pedersen et al. 2008; Kapoor et al. 2019), a 4- to 5-fold higher risk in boys than girls (MacMahon 2006), and is the most common disease requiring surgery in the first months of life (Chung 2008). Despite its well-established clinical presentation, diagnosis and treatment, IHPS etiology remains incompletely understood, with both genetic and environmental factors contributing to disease pathogenesis. IHPS familial aggregation of 20-fold increased risk among siblings (Krogh et al. 2010), twin heritability estimates of 87% (Krogh et al. 2010) and genome wide SNP heritability of 30% (Fadista et al. 2019) suggest a strong genetic component for IHPS. Furthermore, perinatal risk factors such as exposure to macrolide antibiotics (Lund et al. 2014), being first-born, preterm, delivery by cesarean section (Krogh et al. 2012a.1), and bottle-feeding (McAteer et al. 2013; Krogh et al. 2012b.2) highlight the importance of modifiable early environmental exposures on the risk of IHPS (Zhu et al. 2017).

Previous findings from our group and others showed an association between IHPS and lipid metabolism. Briefly, we detected genetic variants at the *APOA1* locus in which known cholesterol-lowering alleles were associated with increased risk of IHPS (Feenstra et al. 2013). Moreover, in the same study we found lower levels of total cholesterol in umbilical cord blood in cases vs. controls (Feenstra et al. 2013). In our most recent genome-wide association study (GWAS) meta-analysis of IHPS, we also detected a genome-wide positive genetic correlation with high-density lipoprotein (HDL) cholesterol and a negative correlation with very low-density lipoprotein cholesterol (VLDL) (Fadista et al. 2019). Moreover, 14% of children with Smith–Lemli–Opitz syndrome (SLOS), a malformation syndrome caused by an inborn defect of cholesterol biosynthesis (Tint et al. 1994), are reported to have IHPS (Kelley and Hennekam 2000).

Following on these previous findings, this study had two goals. The first goal was to investigate associations between IHPS and lipid-related metabolites in the blood of neonates from dried blood spot samples obtained through the routine Danish neonatal screening program (Nørgaard-Pedersen and Hougaard 2007). The second goal was to address the question of whether detected differences in metabolite levels were likely to be driven by genetic differences between IHPS cases and controls or by differences in early life feeding patterns, i.e. breastfeeding vs. formula-feeding or feeding difficulties in the first days of life.

## Methods

### Study participants

Study participants were born between 1997 and 2014 and belong to a Danish ancestry cohort previously described in our genome-wide meta-analysis of IHPS (Fadista et al. 2019). Briefly, whole blood from heel prick sampling of newborns was blotted on filter paper and stored as dried blood spot (DBS) samples as part of the routine Danish neonatal screening program. DBS samples from children born before 2009 had a median age of sampling of 6 days (5–8 days; 1st–3rd quartiles), while children born from 2009 onwards had a median age at sampling of 2 days (2–3 days; 1st–3rd quartiles) (Supplemental Fig. 1). IHPS cases were defined as having a pyloromyotomy (registered in the Danish National Patient Register (Lynge et al. 2011)) in their first year of life, were singletons born in Denmark with parents and grandparents born in Northwestern Europe, and were born with no severe pregnancy complications nor major congenital malformations (see Fadista et al. 2019). For controls we used the same selection criteria as for cases, but without any IHPS diagnosis or surgery code. The initial sample size comprised a total of 588 samples (3.2 mm punches) taken from DBS cards (294 pairs of cases and controls matched by sex and day of birth), of which we removed sequentially the following samples: (a) 2 pairs due to low sample quality in the cases; (b) 19 pairs due to at least one of the samples in each pair not passing standard GWAS QC (e.g. genetic variant missingness, ancestry outlier or genetic relatedness) (Fadista et al. 2019); (c) 5 pairs in which the controls had missing parity information; and (d) 1 pair in which the case had missing gestational age information. After this filtering, the discovery cohort comprised 267 pairs of IHPS cases and controls matched by sex and day of birth (range from 1997 to 2014) (Table 1, Supplemental Fig. 2). The 267 IHPS cases represent a random 1/3 of all surgery confirmed cases in Denmark in the first year of life during the study period of 1997 up to 2014 (http://biobanks.dk/). All 534 samples were selected from DBS samples taken a few days after birth and stored in the Danish Neonatal Screening Biobank (Nørgaard-Pedersen and Hougaard 2007). Sex, date of birth, gestational age, maternal age, Apgar scores (a standardized assessment score for infants after delivery) and parity information were extracted from the Danish Medical Birth Register (Bliddal et al. 2018), while diagnosis and surgery codes were extracted from the Danish National Patient Register (Lynge et al. 2011). Information on DBS sampling days after birth was available since 2006 in the Danish Neonatal Screening database. Sample summary characteristics are described in Table 1. This study was approved by the Danish Scientific Ethics Committee and the Danish Data Protection Agency. An exemption from obtaining informed consent from participants was given as this research project was based on samples from biobank material (H-4-2013-055).

**Table 1:** Study sample characteristics

	Cases (N = 267)	Controls (N = 267)
Boys, no. (%)	233 (87.3)	233 (87.3)
Year of birth (range)	1997–2014	1997–2014
Year of birth, mean (SD)	2008 (4.6)	2008 (4.6)
Maternal age, mean (SD), years	29.8 (5.3)	30.5 (4.8)
Gestational age, mean (SD), weeks	39.8 (1.7)	40.0 (1.4)
Parity, mean (SD)	1.7 (1.0)	1.8 (0.8)
Apgar scores^a^, mean (SD)	9.8 (0.7)	9.9 (0.4)
Age at DBS sampling, mean (SD), days	3.3 (2.2)	3.2 (1.8)
Age at diagnosis, mean (SD), days	36.8 (18.1)	
Breastfeeding difficulty (P92.5), no. (%)	17 (6.4)	4 (1.5)
Cesarean section, no.	57 (21.3)	45 (16.9)

^a^The Apgar score is a standardized assessment for infants after delivery. It is based on a total score of 1 to 10, with the higher the score, the better the newborn is doing after birth

### Metabolite profiling and nomenclature

The DBS samples consist of whole blood blotted onto filter paper, dried at room temperature for at least 3 h before being sent by mail for newborn screening at Statens Serum Institut. After newborn screening, the samples were then stored at − 20 °C in the Danish National Biobank (Nørgaard-Pedersen and Hougaard 2007). On the day of the analysis, we collected a 3.2-mm punch using a Panthera-PuncherTM 9 blood spot punching system (PerkinElmer, Waltham, MA, USA) directly into the 96-well kit plates.

Targeted metabolite quantification followed the manufacturer’s user manual for the AbsoluteIDQ® p400 Kit (Biocrates Life Sciences AG, Innsbruck, Austria), adapted for DBS analysis by the manufacturer (Biocrates 2018.1). The kit has a high-performance liquid chromatography (LC) separation step and a flow injection analysis (FIA) step, both followed by mass spectrometry (MS) analyses. Mass detection and compound identification are performed by multiple reaction monitoring. The method of the AbsoluteIDQ® p400 kit gives accurate results with good inter-laboratory precision for serum and plasma analysis (Thompson et al. 2019). Moreover, it has comparable results to the AbsoluteIDQ® p180 kit (Biocrates 2018.2), a kit with reported high interlaboratory reproducibility (Siskos et al. 2017), and validated for human blood plasma and DBS (Zukunft et al. 2013). The AbsoluteIDQ® p400 kit allows simultaneous quantification of 408 metabolites comprising 55 acylcarnitines, 21 amino acids, 21 biogenic amines, 196 glycerophospholipids (24 lysophosphatidylcholines and 172 phosphatidylcholines), 40 sphingolipids (31 sphingomyelins and 9 ceramides), hexoses (about 90–95% glucose), 14 cholesterol esters and 60 glycerides (18 diglycerides and 42 triglycerides). Lipid metabolites are abbreviated as follows: acylcarnitines as AC(X:Y); hydroxylacylcarnitines as AC(X:Y-OH); dicarboxylacylcarnitines as AC(X:Y-DC); phosphatidylcholines as PC(X:Y); lysophosphatidylcholines as LPC(X:Y); sphingomyelins as SM(X:Y); ceramides as Cer(X:Y); cholesterol esters as CE(X:Y); diglycerides as DG(X:Y) and triglycerides as TG(X:Y). Each lipid metabolite is mentioned as X:Y, with X being the number of carbon atoms and Y the number of double bonds. Quality was controlled on each plate by the injection of three paper-based blanks and three quality control (QC) levels (QC1-3), of which the medium level (QC2) was injected in five replicates. We calculated absolute concentrations based on 7-point calibration curves for LC-MS metabolites and 1-point calibration for FIA-MS metabolites.

The equipment (Thermo Fisher Scientific, Waltham, MA, USA) consisted of a Combi PAL HTS TMO autosampler, a LX-2 LC system with two injectors, each connected to an Ultimate 3000 Dionex RS pump and to the MS through a Transcend II Valve Interface Module. MS data was acquired on a QExactive with a heated electrospray ionization source. All solvents that were not provided with the kit were Optima™ LC/MS grade and purchased from Thermo Fisher Scientific. We controlled the instruments using TraceFinder 4.1 Clinical Research, Aria MX V2.2 and QExactive tune software V2.8 SP1.

We first injected extracts for LC-MS measurements on one dedicated injector (amino acids, biogenic amines). We conducted FIA the next day on the second dedicated injector (acylcarnitines, glycerophospholipids, sphingolipids, hexoses, cholesterol esters, glycerides). We ran eight 96-well plates, with wells measured in a fixed order (from #1 to #96). A matched case and control would always be on the same plate but not necessarily injected sequentially. We pre-processed LC-MS data using Xcalibur 4.1, and integrated all data in Biocrates MetIDQ Carbon-2793 software for quality assessment, quantification of metabolite concentrations by reference to appropriate internal standards, and to do inter- and intra-plate normalization using the median value of the five QC2 replicated injections of each plate in comparison with their target values (see below).

### Data cleaning and statistical analysis

After quantification, we performed data cleaning of the 408 quantified metabolites, resulting in a reduced set of quality controlled metabolites for the association analyses. For each metabolite m and plate p, we defined the limit-of-detection (LOD_m,p_) as the median non-normalized values of metabolite m for paper-based blanks (done in triplicate for each plate) on the relevant plate p. This means that LODs were calculated separately for each plate. We excluded a metabolite m, if more than 20% of non-normalized concentrations in cases and controls were ≤ LOD. We implemented MetIDQs data normalization procedure in R (R Core Team 2018), to scale out differences between plates. Briefly, we calculated the median value per metabolite per plate of the five replicated injections of the QC2 (A_m,p_) (only for cells above LOD) and divided that by the metabolite target value (TV_m_) of each metabolite, available in the MetIDQ database, leading to the correction factor by plate (A_m,p_/TV_m_ = C_m,p_). Then to normalize the data, we divided the raw concentration values of all samples by the plate-specific correction factor: Norm_m,p_ = Raw_m,p_/C_m,p_. We also did metabolite filtering by coefficient of variation (CV) based on the normalized values of the QC2 replicates. For each metabolite m, we calculated its CV for all plates together, i.e. for 5 QC2 samples and 8 plates, the CV was based on 40 observations. If CV_m_ > 25% we excluded metabolite m. Thus, of the 408 metabolites measured with the kit, we therefore selected 148 metabolites that were above the limit of detection (LOD) in at least 80% of the samples (in cases or controls separately) and had a coefficient of variation of the QC2 replicates below 25%.

Due to the non-normal distribution of metabolite concentrations (reported in µM), we quantile transformed the normalized metabolite concentrations to a standard normal distribution prior to all statistical analyses. We performed estimation of association of metabolite levels with IHPS with a linear mixed-effects model, implemented in the lmer function from the lme4 R package (Bates et al. 2015), adjusted for sex (as factor), year of birth (as factor), parity, and gestational age (in weeks) as fixed effects, and IHPS case–control pair as a random effect. We defined a statistically significant association as P < 0.05/148, to adjust for the 148 metabolites tested (Bonferroni correction). We also performed a sensitivity analysis adding cesarean section (as factor) to the model.

Since the year 2009 marked a change of policy of the Danish neonatal screening program regarding age at sampling of DBS samples, from a median of 6 days to 2 days after birth (Supplemental Fig. 1), we also performed stratified association analyses for individuals born before 2009 and for individuals born from 2009 onwards, respectively.

### Diagnosis codes and co‐occurrence with IHPS

We extracted all International Classification of Diseases (ICD) diagnosis codes from the 267 pairs of IHPS cases and controls from the Danish National Patient Register (Lynge et al. 2011). We used Fisher’s exact test for count data implemented in R (R Core Team 2018) to calculate the odds-ratio of occurrence of the ICD-code P92.5 (“Neonatal difficulty in feeding at breast”) in IHPS cases vs. controls. We estimated the association of PC(38:4) levels with occurrence of ICD-code P92.5 at day of birth vs. after day of birth with a linear model, implemented in the lm function from R (R Core Team 2018), adjusted for sex (as factor), year of birth, parity, and gestational age (in weeks) and IHPS status.

### Genetic variants associated with metabolite levels

Since *FADS1* SNP rs174547 is known to explain an appreciable proportion of PC(38:4) variance (11%) in adults (Draisma et al. 2015), we chose it as an instrumental variable to test for association between genetically determined levels of PC(38:4) and IHPS risk. We assessed the association of *FADS1* genetic variant rs174547 (Gieger et al. 2008; Suhre et al. 2011; Draisma et al. 2015) with IHPS based on the latest genome-wide meta-analysis of IHPS (Fadista et al. 2019). Since we previously detected genetic variants at the *APOA1* locus in which known cholesterol-lowering alleles were associated with increased risk of IHPS (Feenstra et al. 2013), we estimated the association of the sentinel SNP (rs12721025) for IHPS at the *APOA1* locus (Feenstra et al. 2013; Fadista et al. 2019) against all 148 metabolites. We determined the association of rs174547 (*FADS1*) and rs12721025 (*APOA1*) with metabolite levels in a linear model adjusted for IHPS status, sex, year of birth (as factor), parity, and gestational age (in weeks). We also conducted stratified analysis separately for individuals born from 2009 onwards (lower age at sampling) and for individuals born before 2009 (higher age at sampling).

## Results

### Description of the cohort

We sampled a cohort comprising 267 pairs of IHPS cases and controls matched by sex and day of birth from DBS samples taken a few days after birth (Sect. 2). Key characteristics of the cohort are described in Table 1 and Sect. 2.

### Targeted metabolomics and IHPS association

**Fig. 1:**
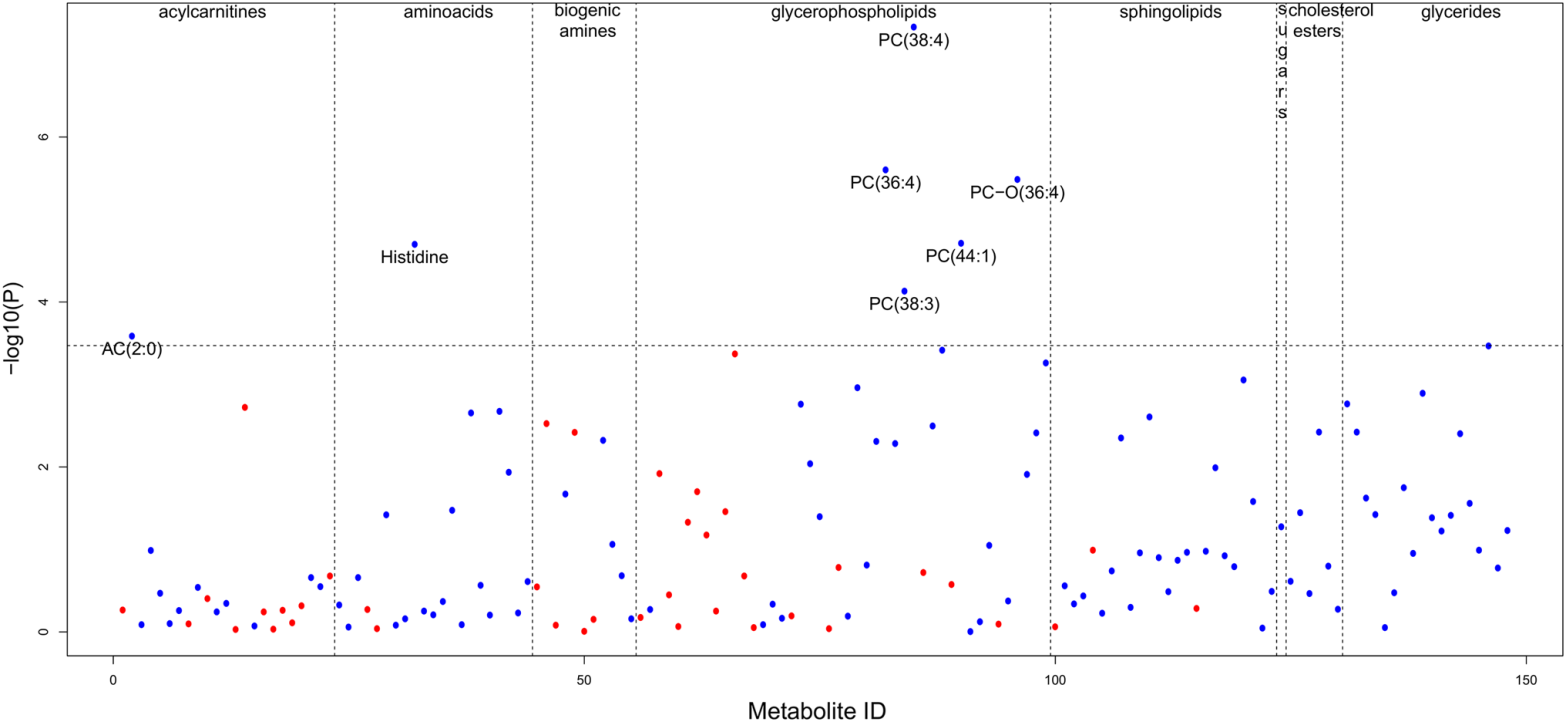
IHPS association with 148 metabolites tested. Dashed vertical lines separate different metabolite classes. The dashed horizontal line marks the Bonferroni-adjusted significance level of P = 0.05/148. Blue and red dots represent metabolites with lower and higher levels in IHPS cases vs. controls, respectively. Case/control normalized concentration fold-changes are between 0.67 and 1.26

**Fig. 2:**
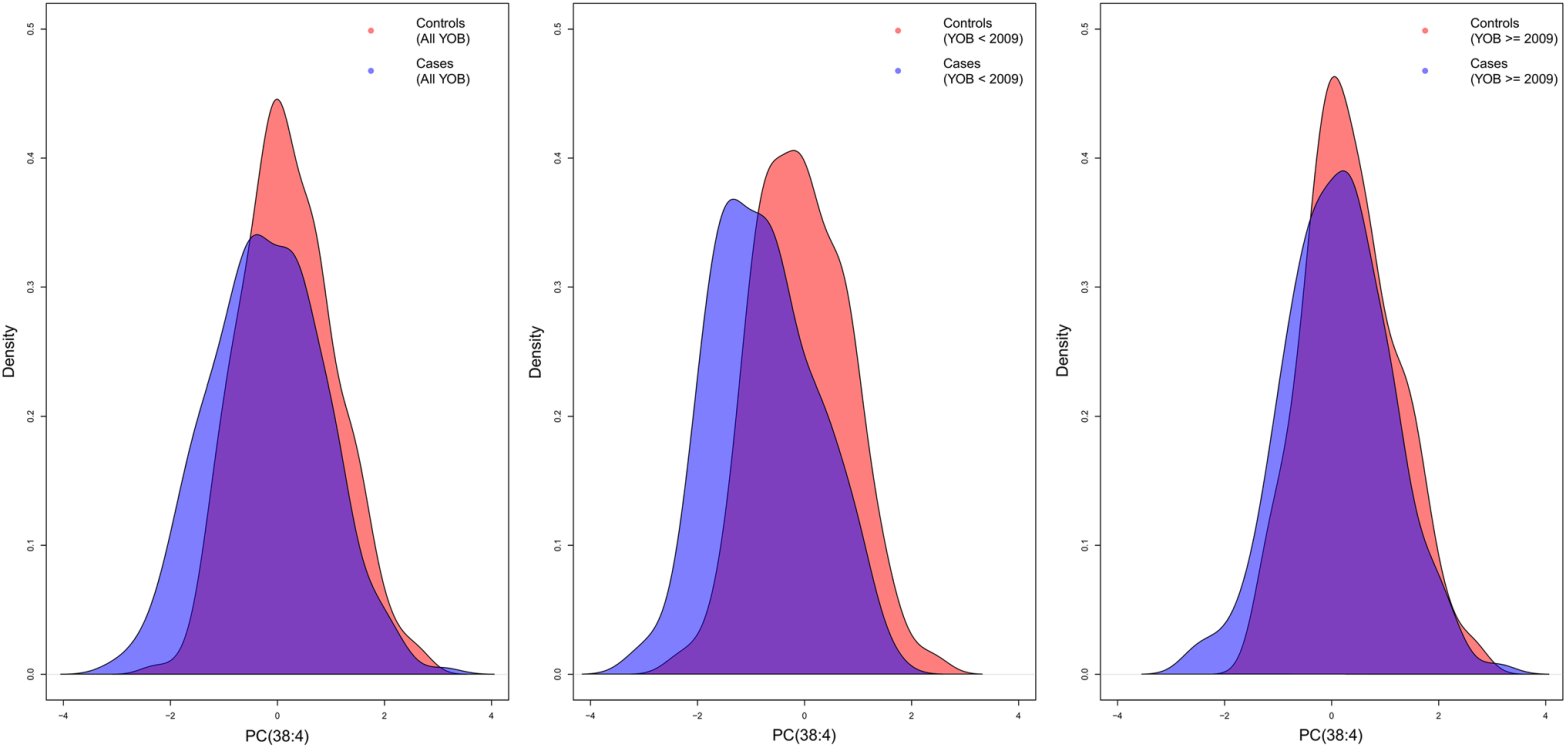
Density plot of the distribution of the quantile transformed normalized concentration of PC(38:4) in our cohort of 267 pairs of IHPS cases and controls

**Fig. 3:**
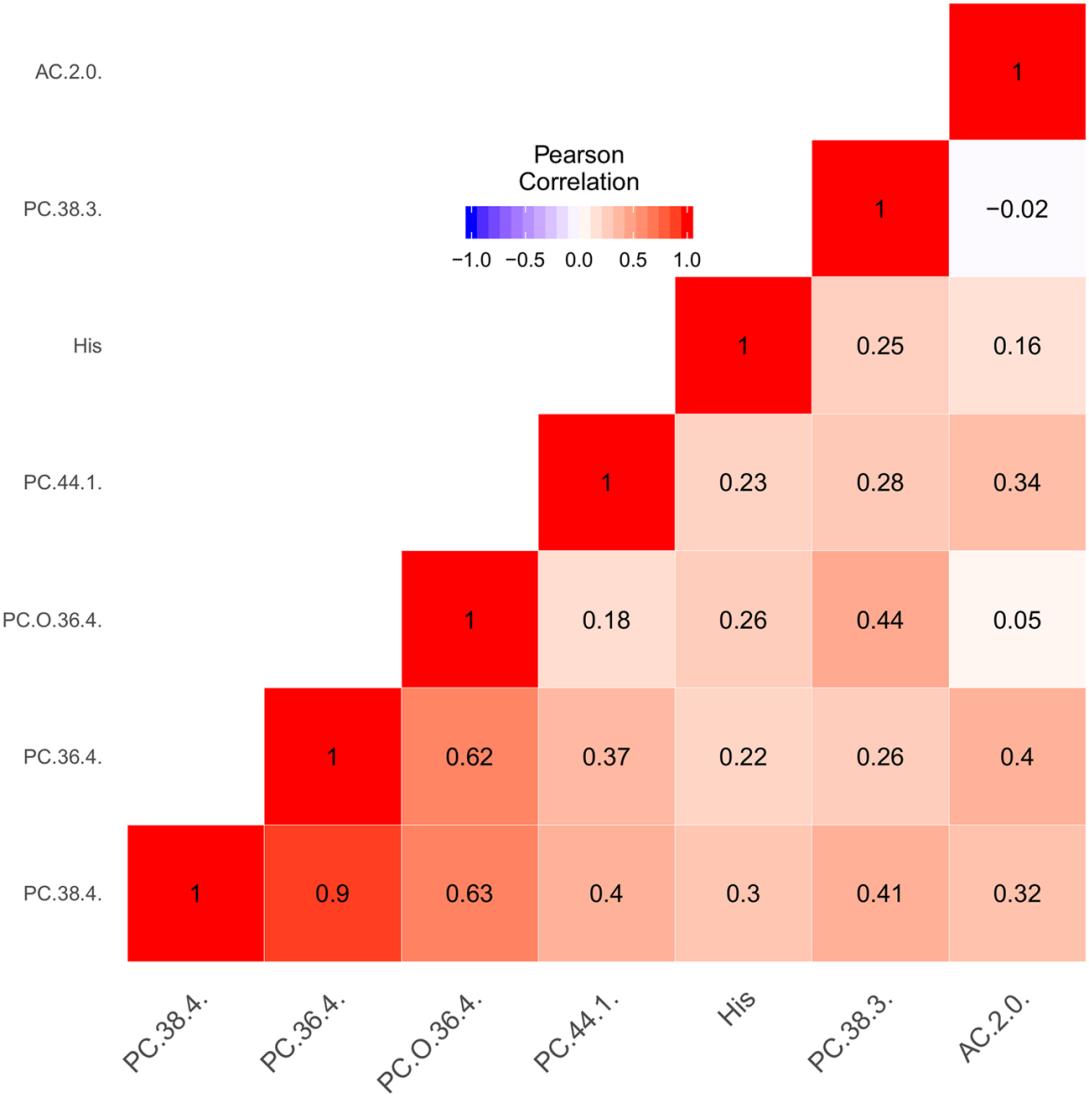
Pairwise complete Pearson correlation between the quantile transformed normalized concentrations of the seven significantly IHPS associated metabolites

We chose a lipid-centric targeted quantitative metabolomics platform for metabolic profiling of IHPS cases and controls (Sect. 2). Of the 408 metabolites measured with the Biocrates p400 Kit, 148 metabolites passed data cleaning and quality control (Sect. 2). Using a mixed-effects linear regression model (Sect. 2), seven of the 148 metabolites showed significantly lower levels in IHPS cases (Table 2; Fig. 1, Supplemental Table 1). The seven metabolites included acylcarnitine AC(2:0), the amino acid histidine and five phosphatidylcholines (PC(38:4), PC(36:4), PC-O(36:4), PC(44:1) and PC(38:3)) (Table 2; Fig. 1, Supplemental Figs. 3–8). The top IHPS associated metabolite was phosphatidylcholine PC(38:4) (P = 4.68 × 10^−8^) (Fig. 2), while the concentrations of the remaining six metabolites showed significant positive correlation with PC(38:4) (all correlations with P < 2.30 × 10^−12^; test for correlation between paired samples using Pearson’s product moment correlation coefficient) (Fig. 3). After correction for multiple comparisons, we only detected significant association of metabolite levels with IHPS for individuals born before 2009 but not from 2009 onwards (Table 2; Fig. 2, Supplemental Tables 2 and 3). We did not find evidence of association between sex and levels of PC(38:4) (sex effect in full model described above: P = 0.59) (Supplemental Fig. 9, Supplemental Table 4). Levels of the other six IHPS associated metabolites were also not associated with sex when correcting for multiple testing (Supplemental Table 4). Moreover, there was also no evidence of association between gestational age, parity or cesarean section and levels of PC(38:4) when correcting for multiple testing (Supplemental Table 4).

**Table 2:** Associations of metabolites with IHPS. Results are shown for all pairs and stratified into pairs with year of birth < 2009 or ≥ 2009

Metabolites	267 IHPS case/control pairs (All YOB)			98 IHPS case/control pairs (YOB < 2009; median age = 6 d)			169 IHPS case/control pairs (YOB ≥ 2009; median age = 2 d)		
	Beta	SE	P	Beta	SE	P	Beta	SE	P
PC(38:4)	− 0.42	0.07	**4.68E−08**	− 0.71	0.12	**1.28E−08**	− 0.25	0.09	9.35E−03
PC(36:4)	− 0.36	0.07	**2.51E−06**	− 0.59	0.13	**1.46E−05**	− 0.23	0.09	1.51E−02
PC-O(36:4)	− 0.33	0.07	**3.29E−06**	− 0.68	0.12	**3.44E−07**	− 0.13	0.08	1.10E−01
PC(44:1)	− 0.33	0.08	**1.94E−05**	− 0.44	0.13	1.08E−03	− 0.28	0.10	3.95E−03
Histidine	− 0.34	0.08	**2.01E−05**	− 0.57	0.14	**9.25E−05**	− 0.22	0.09	2.17E−02
PC(38:3)	− 0.32	0.08	**7.39E−05**	− 0.59	0.12	**5.31E−06**	− 0.16	0.10	1.34E−01
AC(2:0)	− 0.25	0.07	**2.60E−04**	− 0.39	0.10	**1.49E−04**	− 0.17	0.09	6.42E−02

Metabolites are ordered by P value in the main analysis including all birth years.. The effect size Beta is with respect to the quantile transformed normalized concentration levels of each metabolite (Sect. 2). P values of the metabolite associations significant after Bonferroni correction are highlighted in bold. Metabolite nomenclature is available in Sect. 2

*YOB* year of birth, *SE* standard error, *P* P value of the association, *d* days

### IHPS-associated metabolites may tag different feeding patterns between cases and controls

In adults the genetic variant rs174547, which is intronic in the *FADS1* gene, has been reported to explain 11% of the variance in PC(38:4) levels (Draisma et al. 2015). In the neonatal cohort analyzed here, rs174547 was associated with the ratio of PC(38:4)/PC(38:3) (P = 5.83 × 10^− 10^) and with PC(38:3) (P = 1.05 × 10^−5^), but not with PC(38:4) (P = 0.30) (Supplemental Fig. 10). For individuals born from 2009 onwards (lower age at sampling), rs174547 was also associated with the ratio of PC(38:4)/PC(38:3) (P = 1.27 × 10^−11^) and with PC(38:3) (P = 2.92 × 10^−6^), but also not with PC(38:4) (P = 0.30) (Supplemental Fig. 11). For individuals born before 2009 (higher age at sampling), rs174547 was not associated with the ratio of PC(38:4)/PC(38:3) (P = 0.17) or PC(38:3) (P = 0.46), nor with PC(38:4) (P = 0.61) (Supplemental Fig. 12). To investigate whether genetically determined levels of PC(38:4) are associated with IHPS, we tested the association of rs174547 with IHPS risk based on the latest IHPS GWAS meta-analysis (Fadista et al. 2019), but no association was found (odds ratio [OR] per rs174547-C allele, 1.04; 95% confidence interval [CI]: 0.95–1.13; P = 0.38) (Supplemental Fig. 13). This suggests that factors other than genetics that influence PC(38:4) levels underlie the association of this metabolite with IHPS risk. Feeding pattern could be one such factor. Although there was no general information on feeding patterns, a larger number of IHPS cases (n = 17) than controls (n = 4) had a diagnosis code for neonatal difficulty in feeding at breast (ICD-10 code P92.5) in the Danish National Patient Register (OR, 4.46; 95% CI: 1.43–18.46; P = 6.15 × 10^−3^) (Table 1, Sect. 2). Furthermore, among the individuals who had the P92.5 code, the ones who received the code already on their day of birth had significantly lower levels of PC(38:4) than individuals receiving the code on a later day (p = 0.036, Supplemental Fig. 14), even after correcting for sex, year of birth, gestational age, parity and IHPS status.

The variant rs12721025 at the *APOA1* locus known to be associated with IHPS (Feenstra et al. 2013; Fadista et al. 2019), was not associated with any of the 148 metabolites after correcting for multiple testing (P > 0.05/148). Furthermore, rs174547 at the *FADS1* locus, was not nominally significantly associated with the seven metabolites associated with IHPS (P > 0.05) (Supplemental Table 5).

## Discussion

In this case–control metabolomics study of IHPS, we identified seven metabolites to have significantly lower levels in newborns who later developed the disease. The metabolites included five PCs, acylcarnitine AC(2:0), and the amino acid histidine, all with concentration levels correlated with each other. The associations were driven by 98 case–control pairs born before 2009, when median age at sampling was 6 days. We did not detect significant associations in the 169 case–control pairs born in 2009 or later, when median age at sampling was 2 days.

Different lines of evidence indicate that our findings may reflect different feeding patterns between the IHPS cases and controls in the first days of life. Serum levels of PC(38:4) and the other associated metabolites are influenced by food intake and genetics. In adults, 11% of the variance in PC(38:4) levels is explained by the genetic variant rs174547 in the *FADS1* gene (Draisma et al. 2015). The *FADS1* gene codes for the fatty acid delta-5 desaturase, a key enzyme in the metabolism of long-chain polyunsaturated omega-3 and omega-6 fatty acids, which catalyzes desaturation of PC(38:3) to PC(38:4) (Gieger et al. 2008; Suhre et al. 2011; Draisma et al. 2015). However, rs174547 was not associated with IHPS in our neonatal cohort, nor was it associated with IHPS in our previous GWAS meta-analysis (Fadista et al. 2019). This suggests that genetically determined levels of the metabolite PC(38:4) are not associated with IHPS risk, requiring another explanation for the observed association between PC(38:4) levels and IHPS. Our data and previous studies point to the importance of feeding patterns. Notably, PC(38:4) is known to be at higher levels in plasma (Uhl et al. 2016) and dried blood spot (Prentice et al. 2015) samples from breast-fed infants compared to bottle-fed infants, and bottle-feeding is a well-known risk factor for IHPS (McAteer et al. 2013; Krogh et al. 2012a, 2012b; Zhu et al. 2017). Although feeding data were not available in our cohort, many more children who developed IHPS than controls had a P92.5 diagnosis code for difficulty in feeding at breast, supporting the hypothesis that a higher proportion of cases were primarily bottle-fed. In addition, newborns who received the P92.5 diagnosis on their day of birth had significantly lower levels of PC(38:4) than individuals with this diagnosis given on a later day further supporting that differences in levels of this metabolite in our data can be attributed to differences in feeding patterns. Finally, it should be noted that right after birth, newborns’ milk intake (whether formula or breast-milk) is very low but increases rapidly within the first week of life (Casey et al. 1986; Dollberg et al. 2001). Thus, the relative importance of feeding pattern variation vs. genetic variation on phosphatidylcholine levels and ratios is likely to increase considerably too. The fact that our associations with IHPS were driven by the smaller subset of newborns (n = 98 pairs) sampled at around 6 days of age and not by the ones (n = 169 pairs) sampled around 2 days after birth is striking in this respect, further supporting that our findings are likely to be attributable to feeding differences between IHPS cases and controls rather than genetic regulation of phosphatidylcholine levels.

While previous work studied the role of bottle feeding as a risk factor for IHPS (McAteer et al. 2013; Krogh et al. 2012b), these have been mainly observational in nature, compiled from health registry data in the first weeks and months after birth. To the best of our knowledge, no previous study has shown data specifically as early as 2 days after birth. It could be that in newborns with genetic susceptibility towards IHPS, the disease risk is slowly potentiated by bottle feeding (or other altered feeding patterns) affecting lipid metabolism, leading to a lag of weeks between birth and the first IHPS symptoms.

Similar to phosphatidylcholines, we also detected histidine and the short-chain acylcarnitine AC(2:0) to be at significantly lower concentrations in IHPS (Fig. 1; Table 2, Supplemental Figs. 6 and 7). Histidine is an essential amino acid (Kopple et al. 1975), i.e. it cannot be synthesized *de novo* in humans and therefore needs to be supplied in the diet. Moreover, histidine content is known to be higher in breast milk compared to standard liquid infant formulas (Agostoni et al. 2000) (preferred formulas in the first days of life). Similarly, it has been reported that infants who are breast-fed have higher concentrations of AC(2:0) in their plasma compared to bottle-fed infants (Kirchberg et al. 2015). These observations are also consistent with the hypothesis that controls were breast-fed to a higher extent than cases.

Further studies are needed to determine whether altered phosphatidylcholines, histidine and acylcarnitine AC(2:0) play a causative role in the pathogenesis of IHPS. It is also possible that these metabolites are just markers of altered feeding patterns, which may exacerbate IHPS risk through other causative pathways. The essential role of phosphatidylcholines in cholesterol metabolism (Cole et al. 2012) warrants further investigation, given our previous findings of lower levels of total cholesterol (TC) in umbilical cord blood of IHPS cases vs. controls (Feenstra et al. 2013), genome-wide genetic correlations of IHPS and cholesterol levels (Fadista et al. 2019), and the reported high incidence of IHPS in children with SLOS (Kelley and Hennekam 2000), caused by a cholesterol biosynthesis defect (Tint et al. 1994). Future studies are required to reveal how genetic and environmental factors may contribute to the pathogenesis of IHPS through disturbances of the lipid metabolism of newborns.

Despite the epidemiological evidence that boys have a 4- to 5-fold higher IHPS risk than girls (MacMahon 2006), and that sex differences exist in serum lipids and lipoproteins at birth (Carlson and Hardell 1977), we did not detect sex differences in the concentrations of the IHPS associated metabolites. The use of small amounts of sample material from dried blood spot samples and the targeted metabolomics approach is a limitation of the study, meaning that relevant metabolites with potential sex differences could have been overlooked. Also, the early sampling time (median of 2 to 6 days after birth) days to weeks before typical occurrence of first IHPS symptoms (2 to 8 weeks after birth) means that potential later differences between the sexes in metabolite levels could have been overlooked.

The study had additional limitations, including the lack of general information on breast-feeding vs. formula feeding so there was no possibility to study the feeding effect directly. In addition, genetic variation known to associate with levels of metabolites queried here is only known in adults, and so genetic associations specific to newborn metabolite levels might have been missed. Strengths of the study include the use of a well-established targeted metabolomics platform (Biocrates) to investigate the association between IHPS and 148 metabolites from DBS samples taken a few days after birth. To our knowledge, this is the first time that extensive newborn metabolomics profiling is being used to study IHPS. Another strength of the study is the use of well-defined codes from nation-wide Danish registers to define outcome, exclusions, and covariates. Finally, the study design in which there was a 1-to-1 matching of IHPS cases and controls based on sex and exact day of birth is also a strength of the study improving efficiency of the analyses.

The observations of this study have implications for ongoing studies of complex diseases in which both genetics and environmental factors play a role in disease etiology. In time, a detailed molecular dissection of IHPS risk factors could potentially be used as a basis for more rapid identification of particularly susceptible newborns. While this study represents a step in that direction, further efforts are needed to replicate these findings in other populations and to better understand the interplay of genetics and early feeding patterns and consequent progression to IHPS. Specifically, the use of wide lipid profiling in nutritional intervention studies could help to unravel causal relationships and underlying mechanisms and optimize the nutritional composition of infant formulas. Moreover, since AC(2:0) is already present on the list of most of newborn screening panels worldwide (McHugh et al. 2011), future work could address the predictive power of AC(2:0) as a newborn screening test for IHPS, and could point to potential preventive and therapeutic targets to those susceptible to IHPS from birth.

## Conclusions

Although IHPS is highly heritable (Krogh et al. 2010; Fadista et al. 2019), it is a complex disease that does not have Mendelian transmission through families. Common genetic variants (Fadista et al. 2019; Feenstra et al. 2013; Feenstra et al. 2012) and environmental risk factors (Lund et al. 2014; Krogh et al. 2012a; McAteer et al. 2013; Krogh et al. 2012b; Zhu et al. 2017) have been previously discovered, but IHPS etiology remains incompletely understood. In this case–control metabolomics study of IHPS, we detected lower levels of PC(38:4), together with correlated PCs, histidine and AC(2:0), in IHPS. Several lines of evidence suggest that environmental factors affecting these metabolites such as feeding pattern in the first days of life may underlie the associations. Further understanding of the complex interplay of genetics and early life environmental factors is critical to the understanding of the pathophysiology, diagnosis and possible treatment or prevention of IHPS, and can serve as a model for other complex diseases.

## Supplementary information

Below is the link to the electronic supplementary material.

Electronic supplementary material 1 (DOCX 16 kb)

Electronic supplementary material 2 (PDF 492 kb)

Electronic supplementary material 3 (PDF 252 kb)

Electronic supplementary material 4 (PDF 250 kb)

Electronic supplementary material 5 (PDF 250 kb)

Electronic supplementary material 6 (PDF 73 kb)

Electronic supplementary material 7 (PDF 225 kb)
