# Supplementary material for: Integrating genetics with newborn metabolomics in infantile hypertrophic pyloric stenosis
Source: Metabolomics. 2021 Jan 8;17(1):7. doi: 10.1007/s11306-020-01763-2 (PMC7794101; doi:10.1007/s11306-020-01763-2)
Supplement: Supplementary file 1 — Electronic supplementary material 1 (DOCX 16 kb) [file 11306_2020_1763_MOESM1_ESM.docx]

**SUPPLEMENTARY TABLE LEGENDS**

**Supplemental Table 1.** IHPS associations for the 148 metabolites tested.

**Supplemental Table 2.** IHPS associations for the 148 metabolites tested (individuals born before 2009).

**Supplemental Table 3.** IHPS associations for the 148 metabolites tested (individuals born ≥ 2009).

**Supplemental Table 4.** Effect estimates of the full model for the seven metabolites associated with IHPS against IHPS case/control status, year of birth (YOB), sex, c-section (cesarean section), gestational age (GA), and parity.

**Supplemental Table 5.** APOA1 SNP rs12721025 associations for the 148 metabolites tested.

**SUPPLEMENTARY FIGURE LEGENDS**

**Supplemental Figure 1.** Boxplots of the distribution of age at sampling (in days) of dried blood spots, stratified by IHPS case/control status and year of birth (YOB) before 2009 and from 2009.

**Supplemental Figure 2.** Participant flowchart.

**Supplemental Figure 3.** Density plot of the distribution of the quantile transformed normalized concentration of Histidine in our cohort of 267 pairs of IHPS cases and controls.

**Supplemental Figure 4.** Density plot of the distribution of the quantile transformed normalized concentration of AC(2:0) in our cohort of 267 pairs of IHPS cases and controls.

**Supplemental Figure 5.** Density plot of the distribution of the quantile transformed normalized concentration of PC(36:4) in our cohort of 267 pairs of IHPS cases and controls.

**Supplemental Figure 6.** Density plot of the distribution of the quantile transformed normalized concentration of PC-O(36:4) in our cohort of 267 pairs of IHPS cases and controls.

**Supplemental Figure 7.** Density plot of the distribution of the quantile transformed normalized concentration of PC(44:1) in our cohort of 267 pairs of IHPS cases and controls.

**Supplemental Figure 8.** Density plot of the distribution of the quantile transformed normalized concentration of PC(38:3) in our cohort of 267 pairs of IHPS cases and controls.

**Supplemental Figure 9.** Boxplots of the distribution of the quantile transformed normalized concentrations of PC(38:4), stratified by sex and IHPS case/control status.

**Supplemental Figure 10.** Boxplots showing the relationship between the genotype of SNP rs174547 and quantile transformed normalized concentrations of PC(38:4), PC(38:3) and the ratio PC(38:4)/PC(38:3).

**Supplemental Figure 11.** Boxplots showing the relationship between the genotype of SNP rs174547 and quantile transformed normalized concentrations of PC(38:4), PC(38:3) and the ratio PC(38:4)/PC(38:3) for individuals born from 2009 onwards (lower age at sampling).

**Supplemental Figure 12.** Boxplots showing the relationship between the genotype of SNP rs174547 and quantile transformed normalized concentrations of PC(38:4), PC(38:3) and the ratio PC(38:4)/PC(38:3).

**Supplemental Figure 13**. Regional association plot of the IHPS association results at the *FADS1* locus. Color-coded linkage disequilibrium is shown for the sentinel SNP rs174547. Linkage disequilibrium determined for the 1000 Genomes EUR population, November 2014 freeze, as described at <http://locuszoom.sph.umich.edu/>. The x-axis represents the genomic region (hg19 assembly) surrounding 400kb of rs174547, while the y-axis represents the strength of the association in −log10(P-value).

**Supplemental Figure 14.** Quantile transformed normalized concentration of PC(38:4) in the individuals that received the ICD code P92.5 ("neonatal difficulty in feeding at breast") at day of birth compared with individuals receiving the ICD code at a later day.
