# Supplementary material for: Integrating genetics with newborn metabolomics in infantile hypertrophic pyloric stenosis
Source: Metabolomics. 2021 Jan 8;17(1):7. doi: 10.1007/s11306-020-01763-2 (PMC7794101; doi:10.1007/s11306-020-01763-2)

Supplemental Figure 1

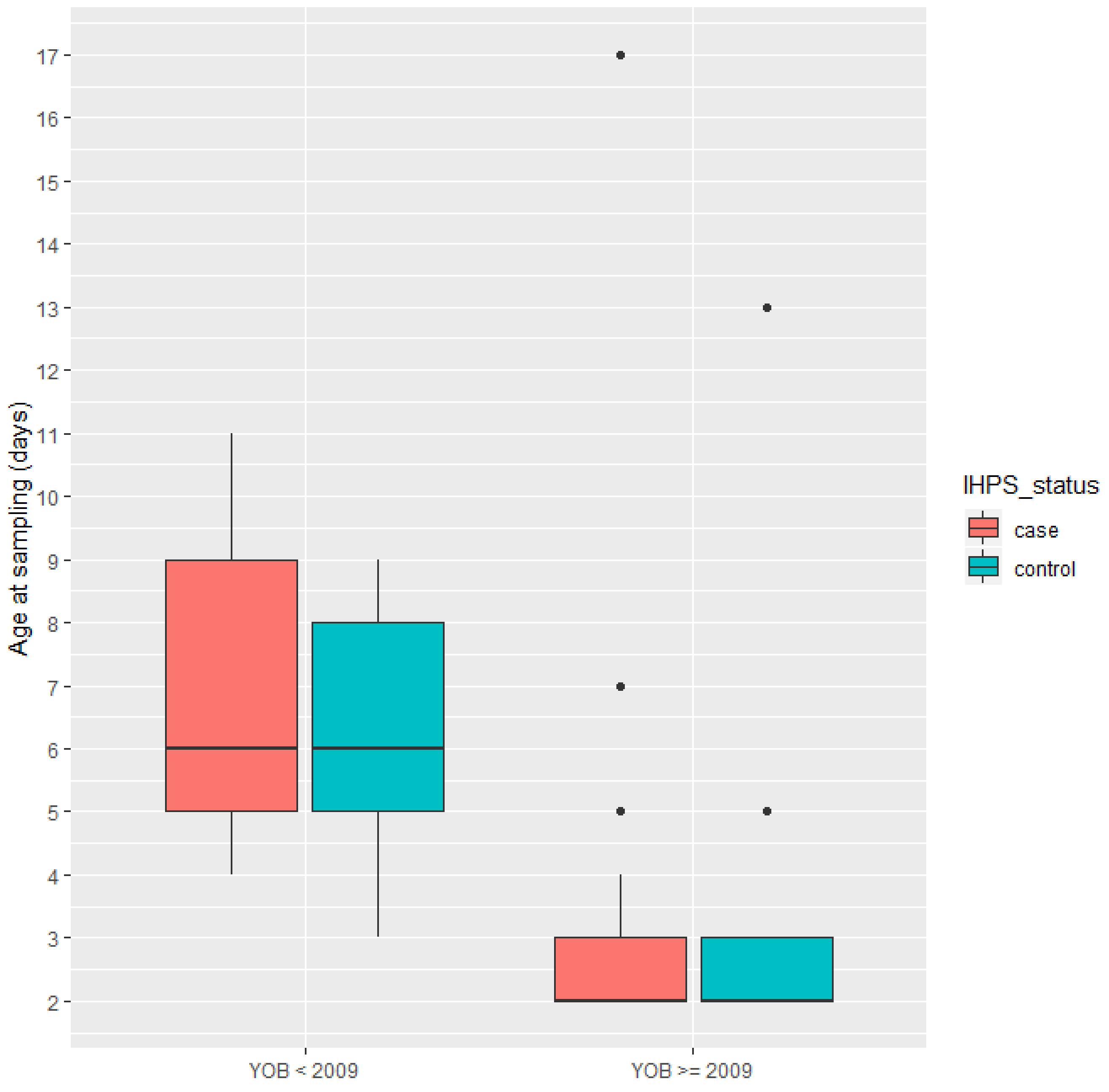

Supplemental Figure 2

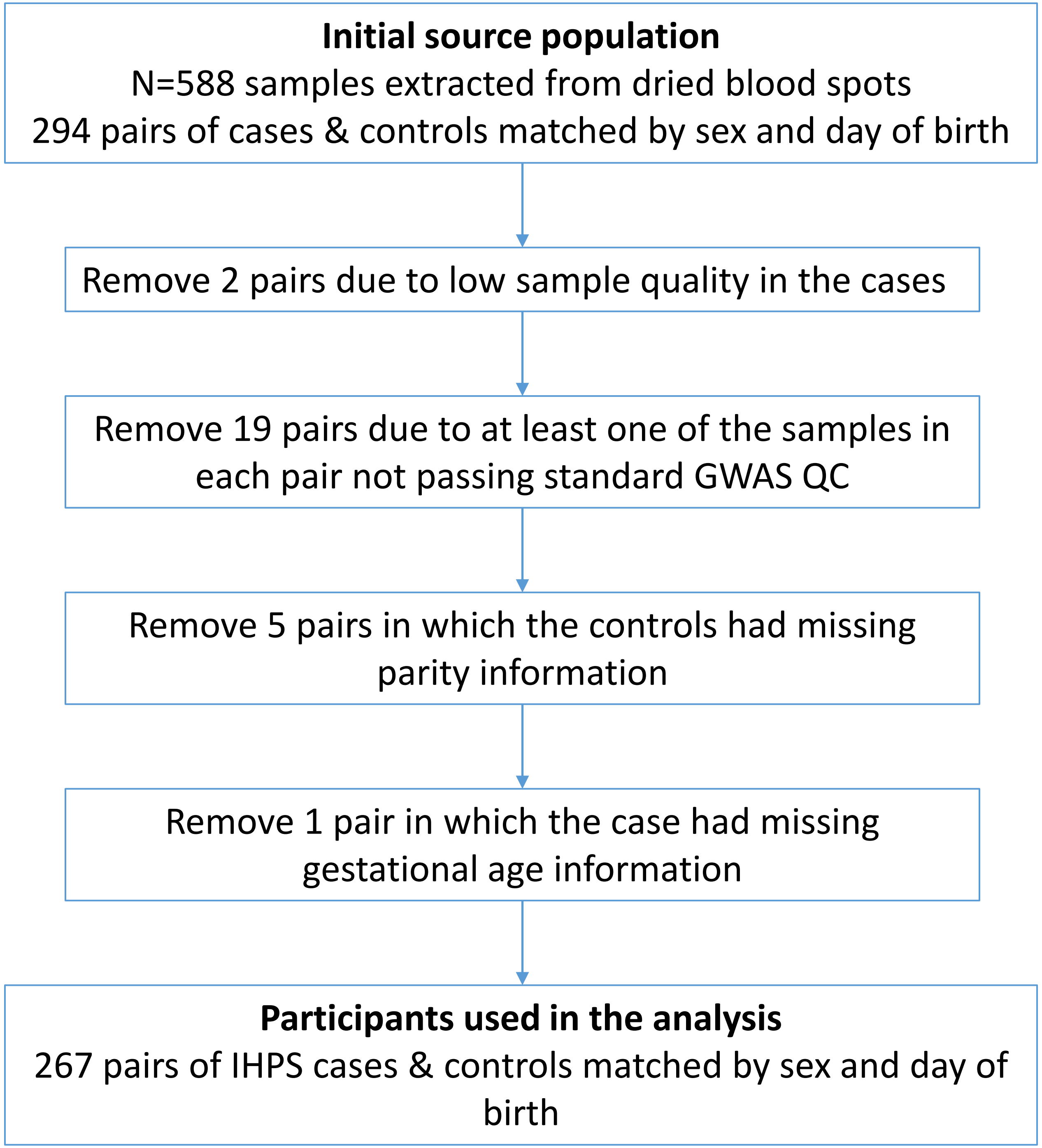

Supplemental
Figure 3

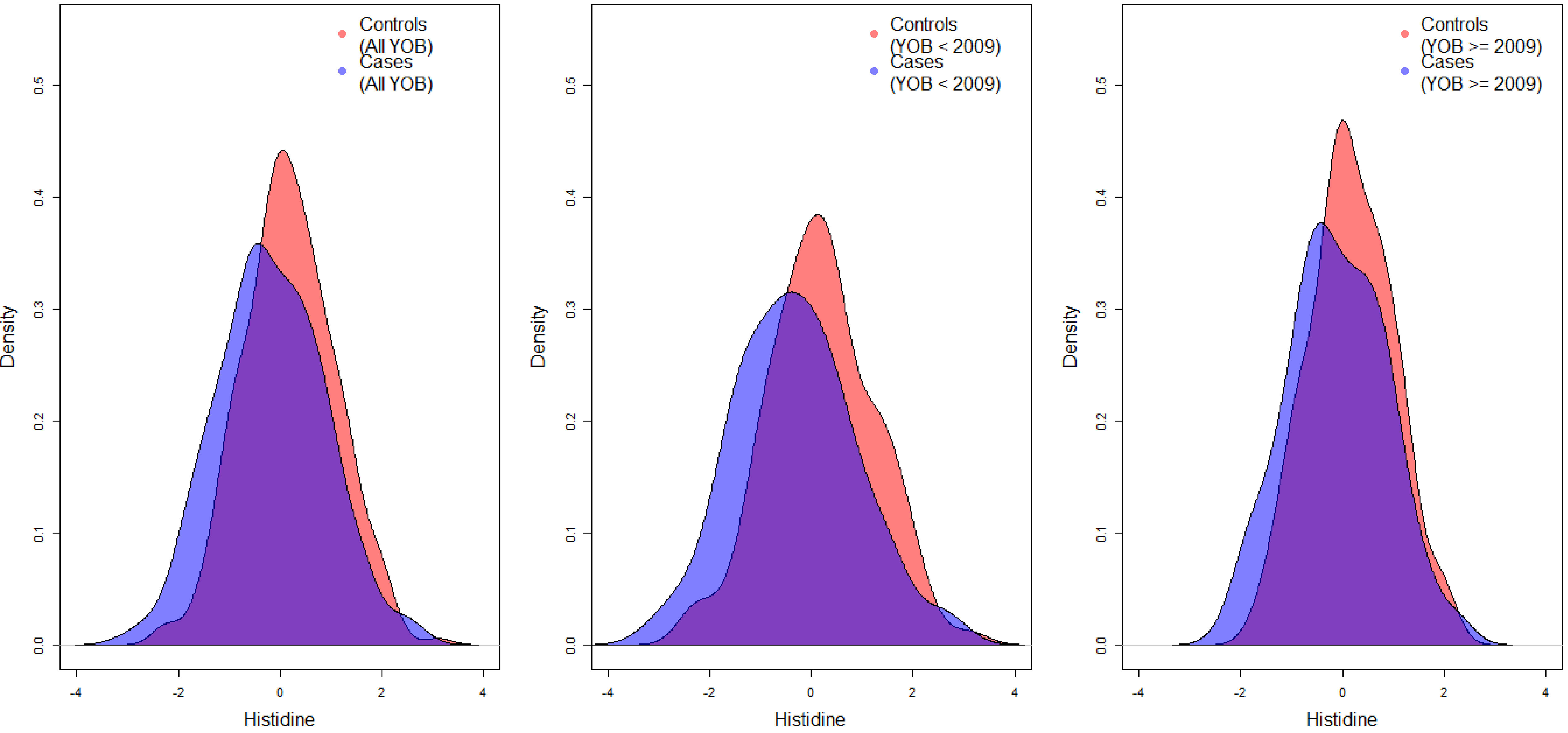

Supplemental
Figure 4

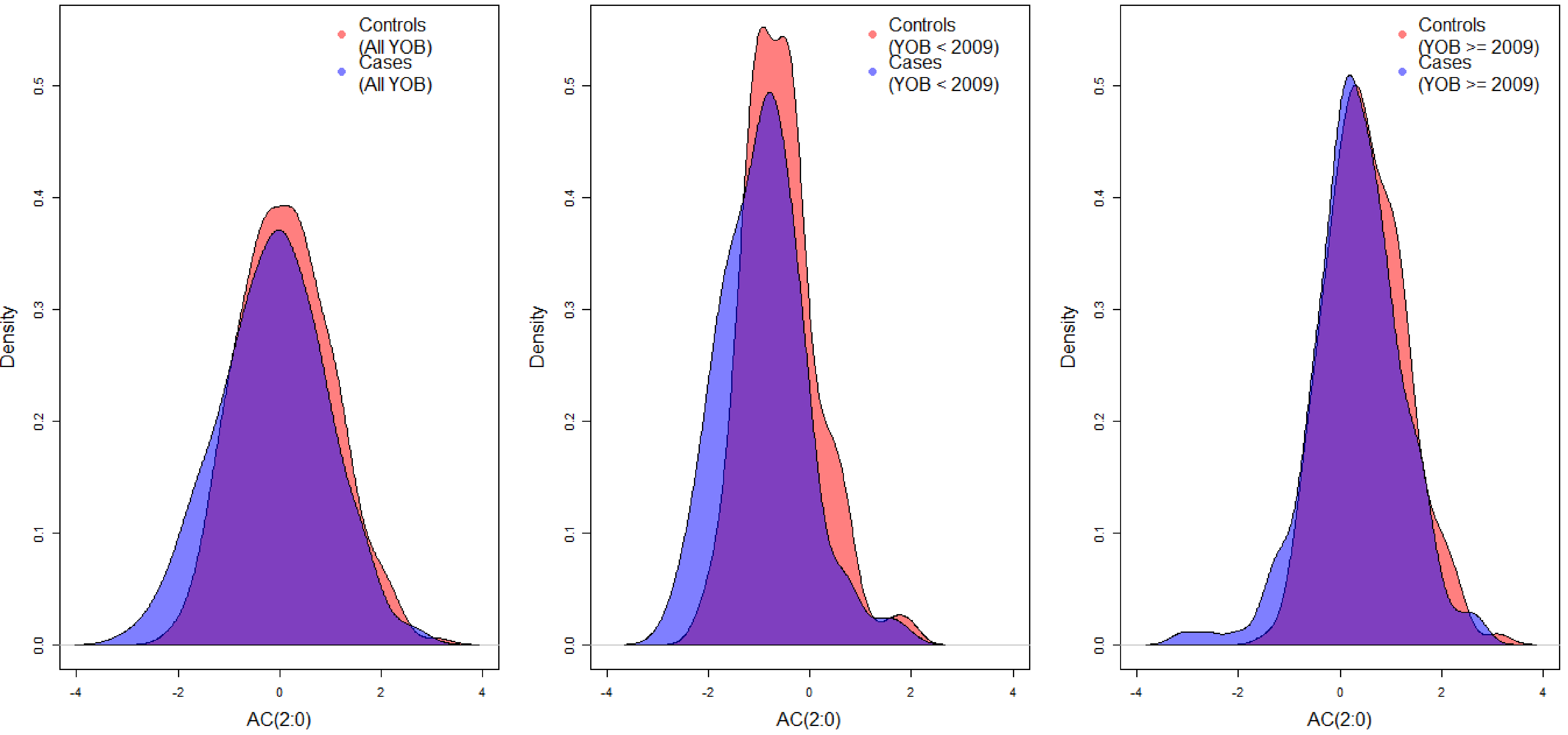

Supplemental
Figure 5

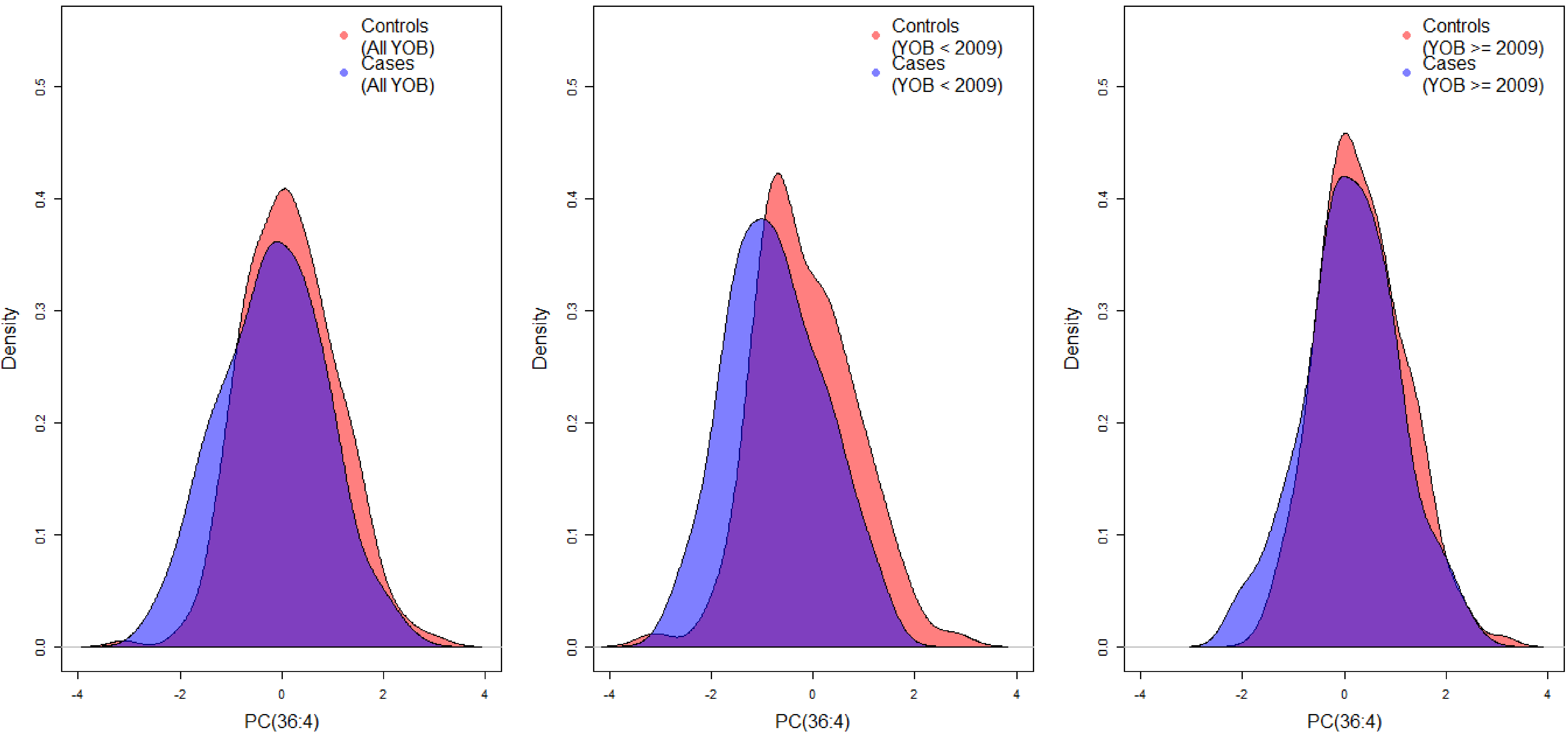

Supplemental
Figure 6

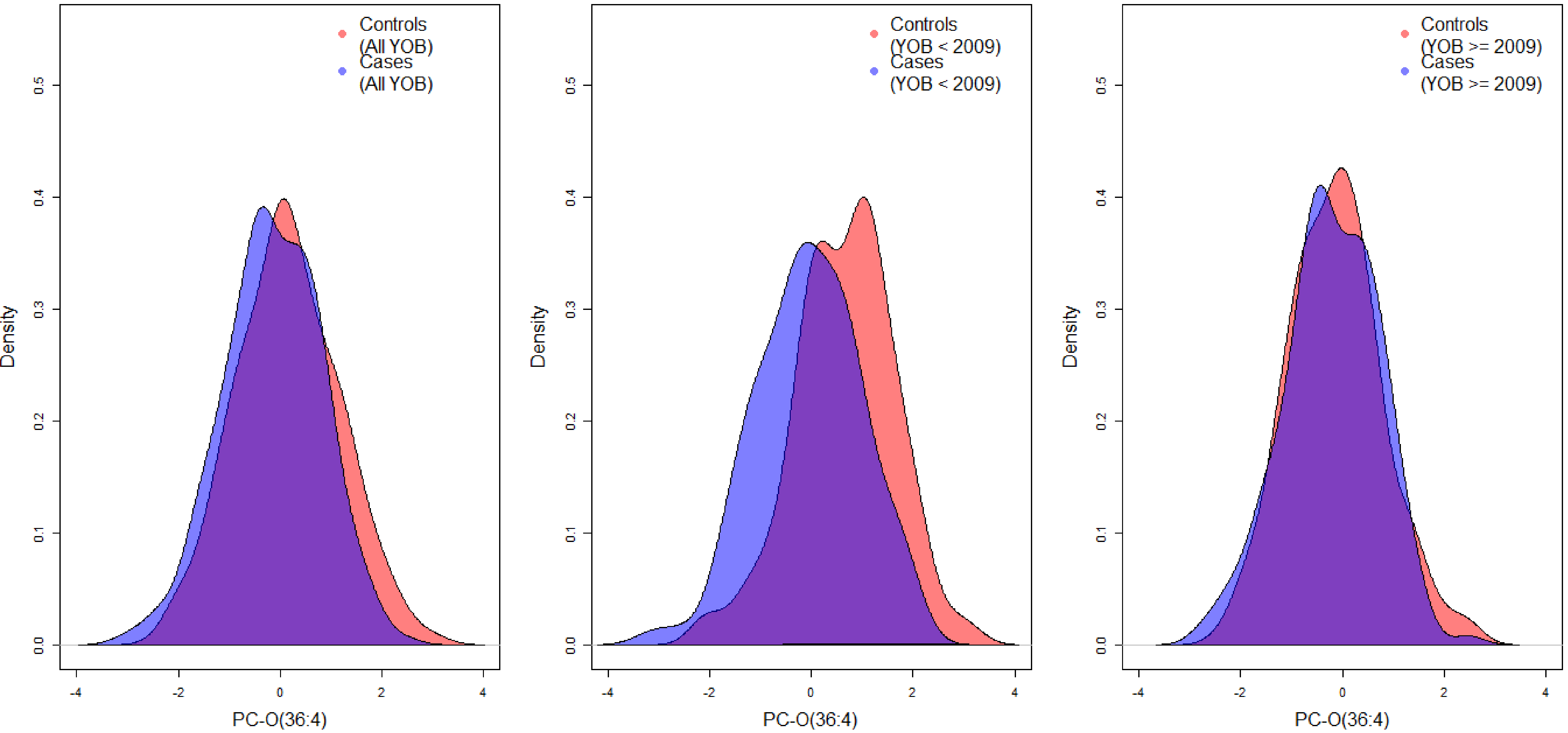

Supplemental
Figure 7

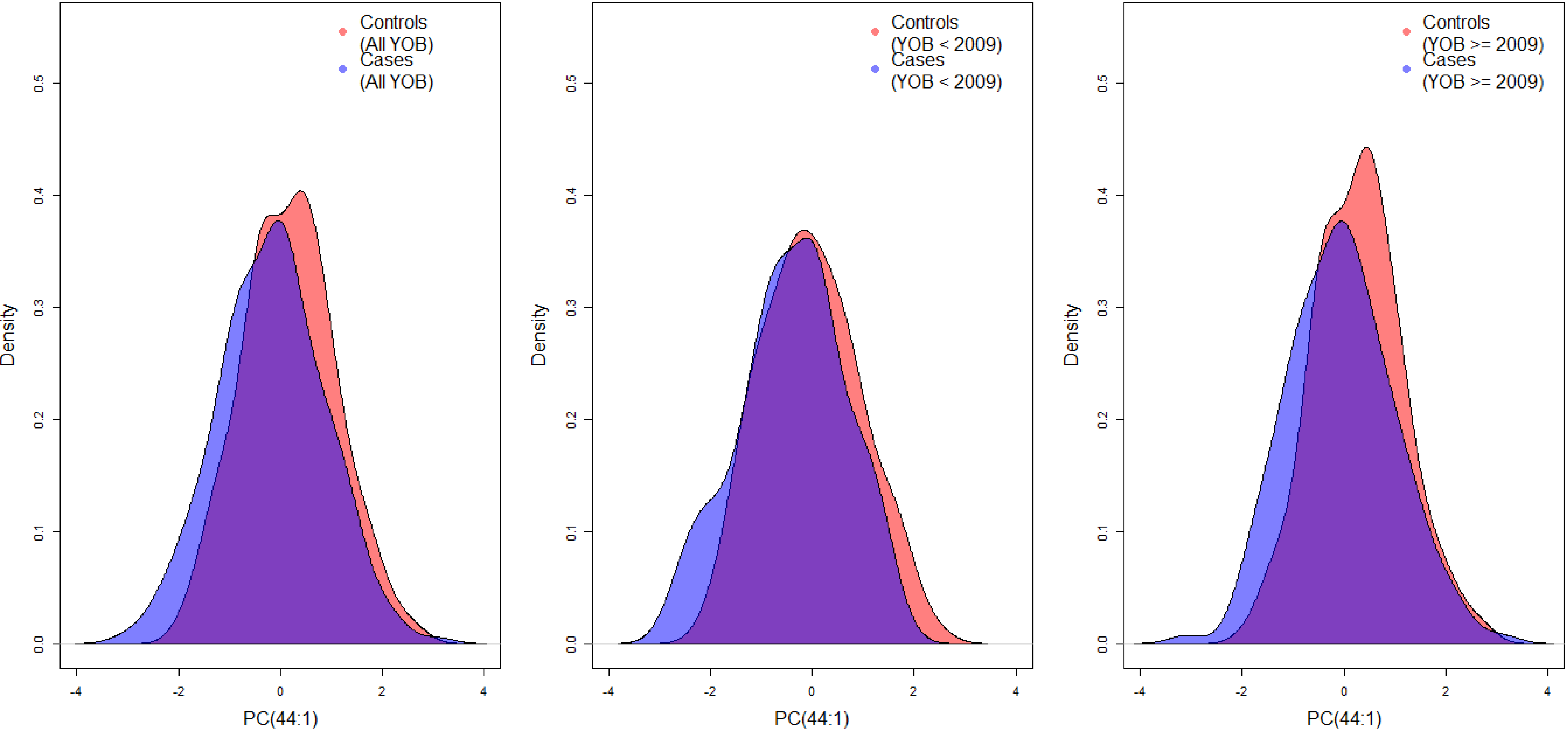

Supplemental
Figure 8

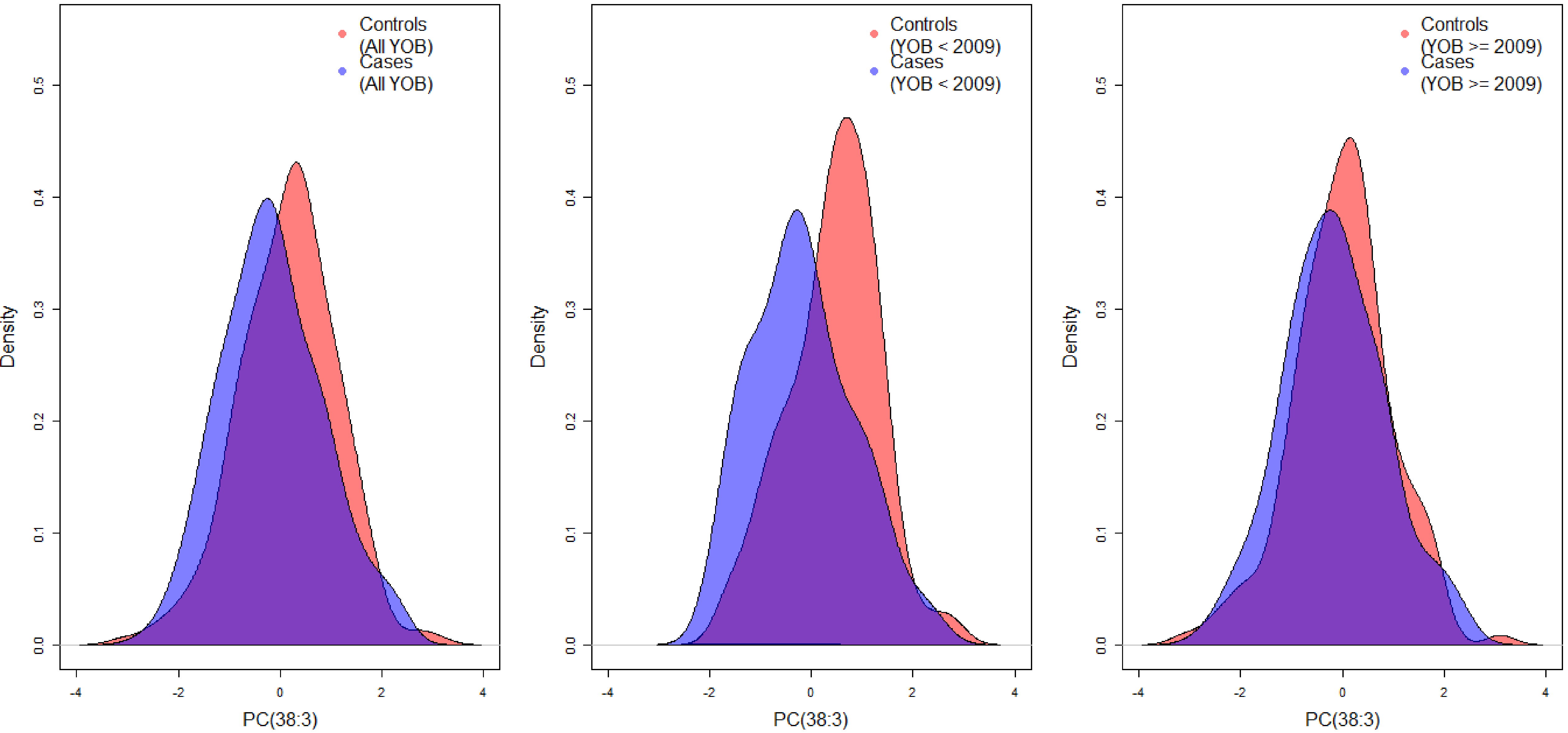

Supplemental
Figure 9

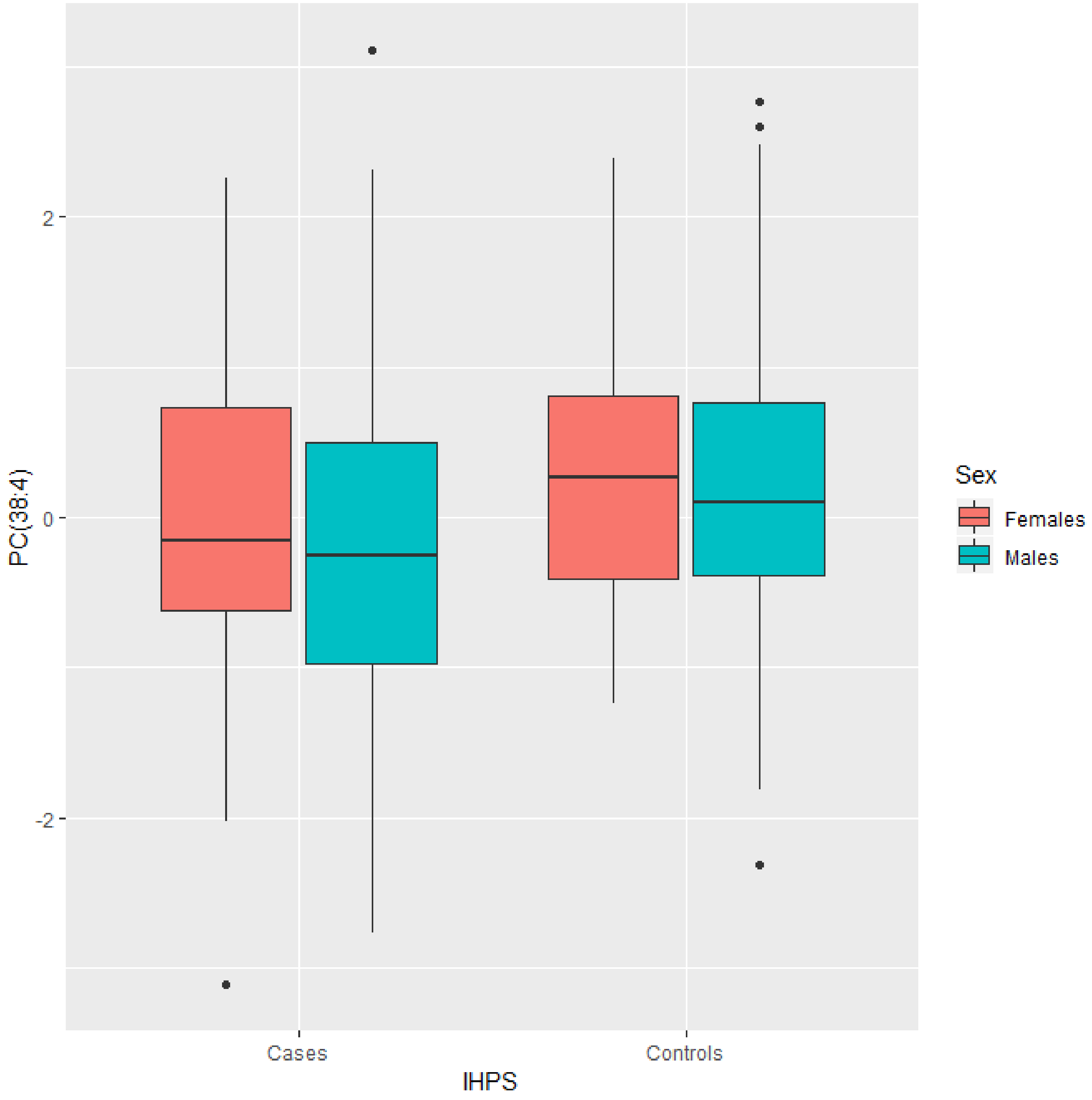

Supplemental
Figure 10

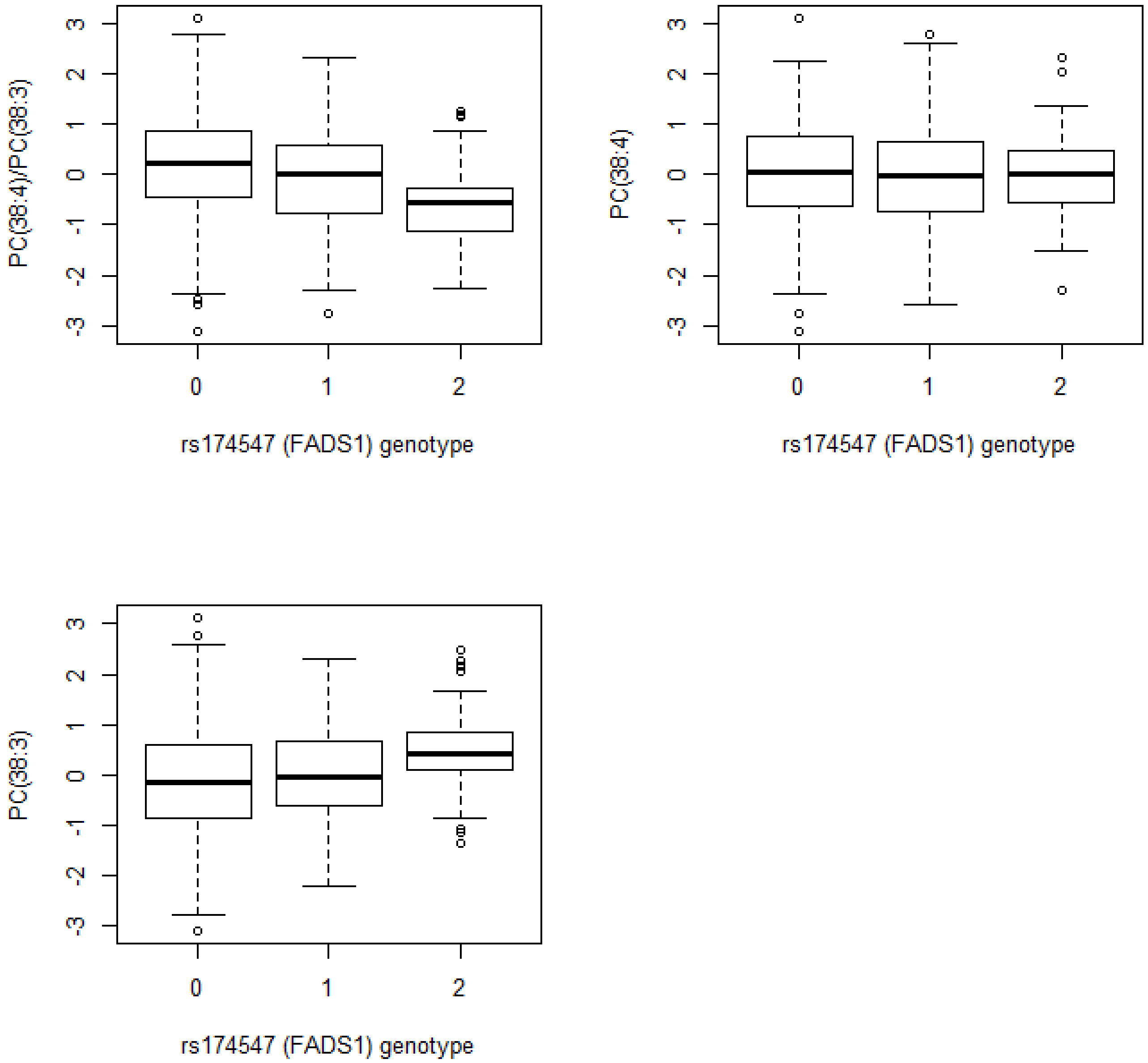

Supplemental
Figure 11

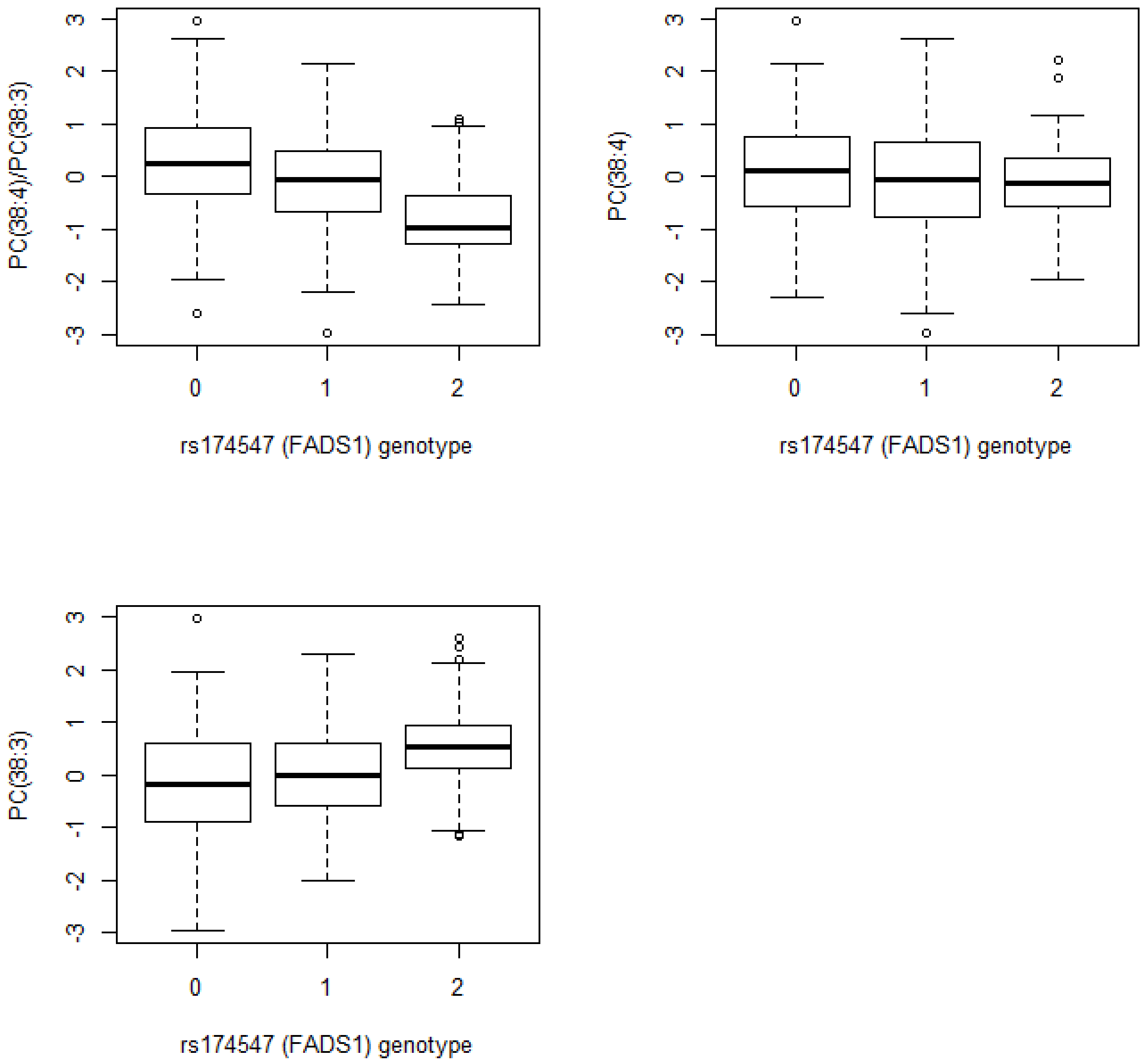

Supplemental Figure 12

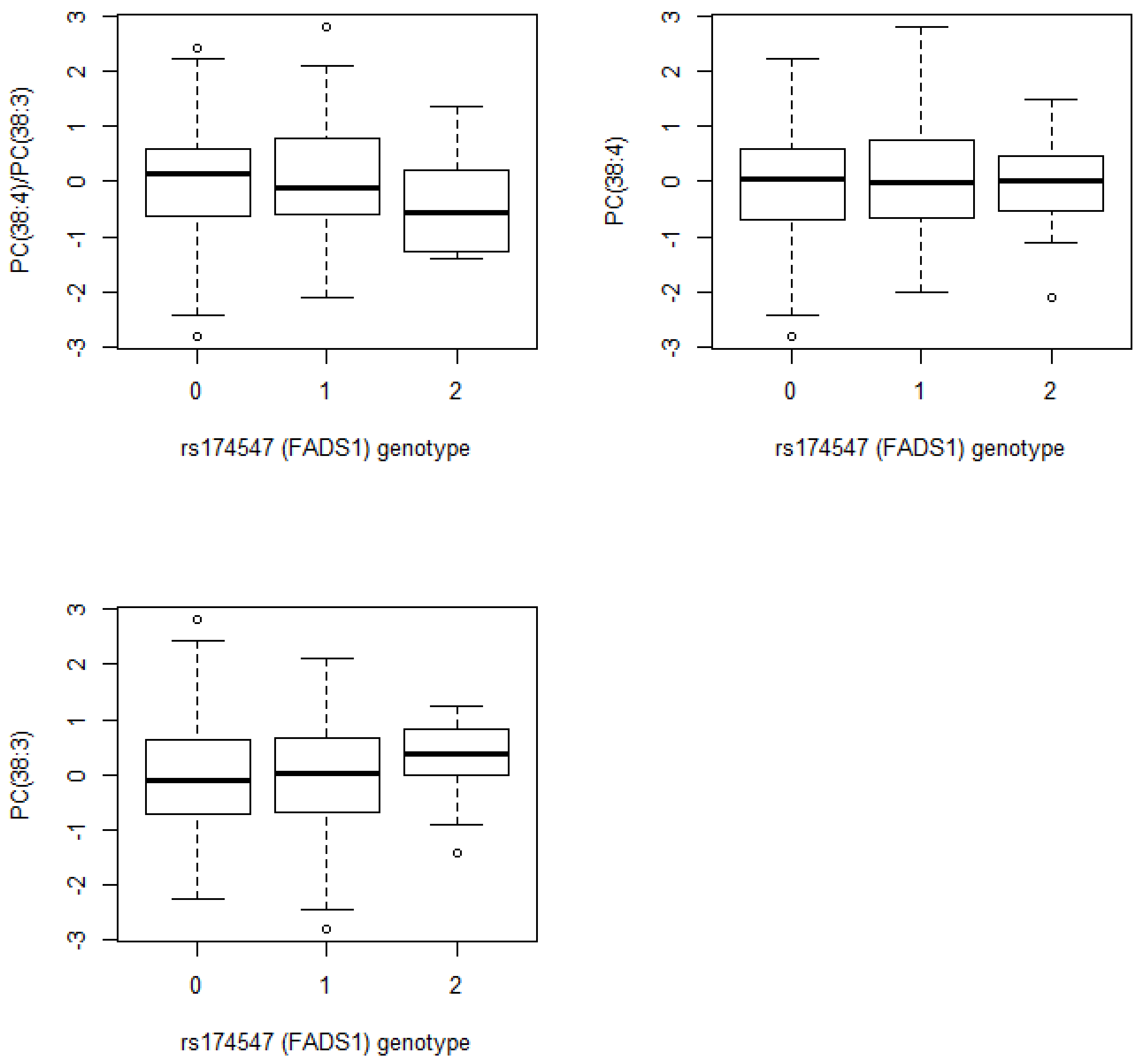

Supplemental Figure 13

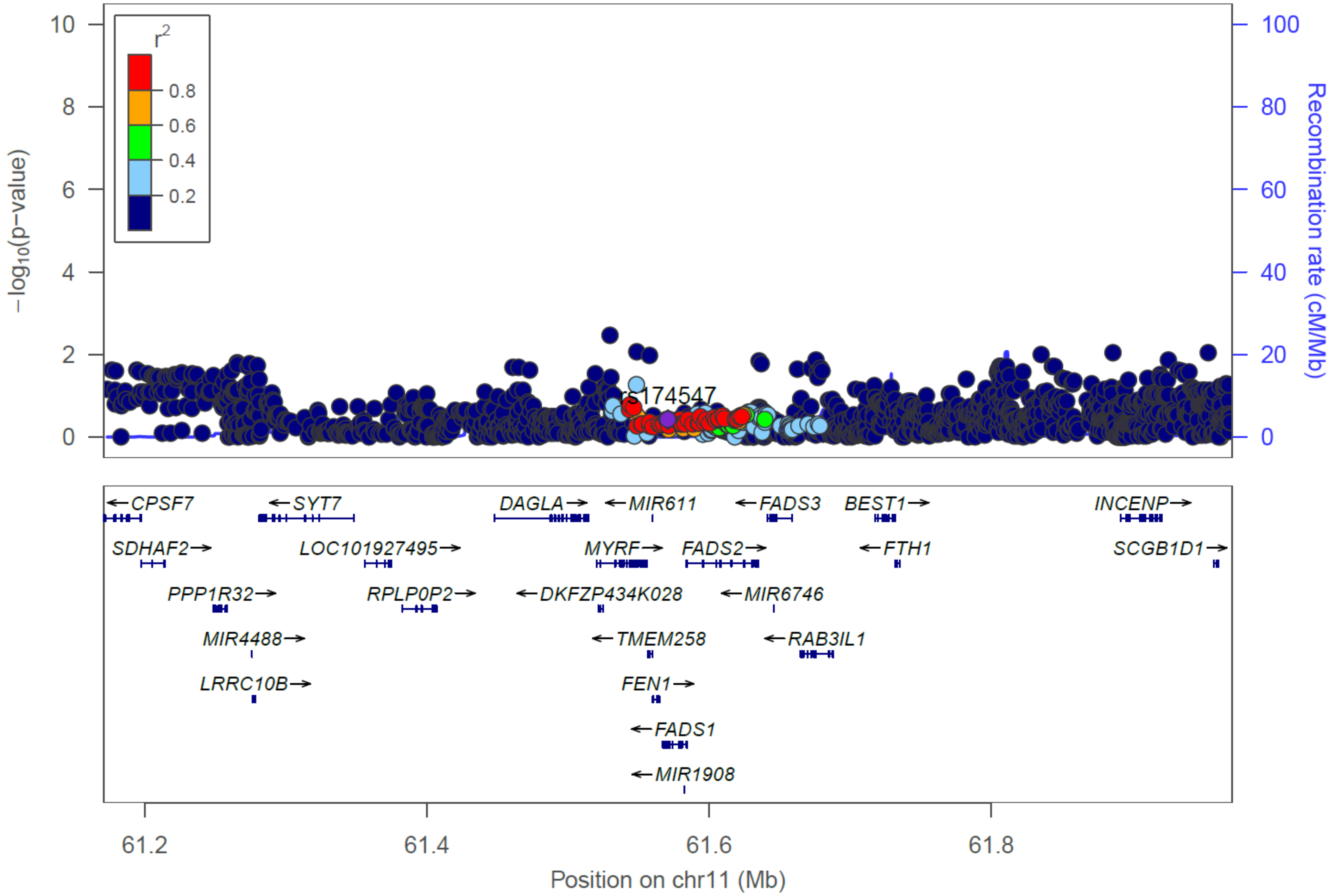

Supplemental
Figure 14

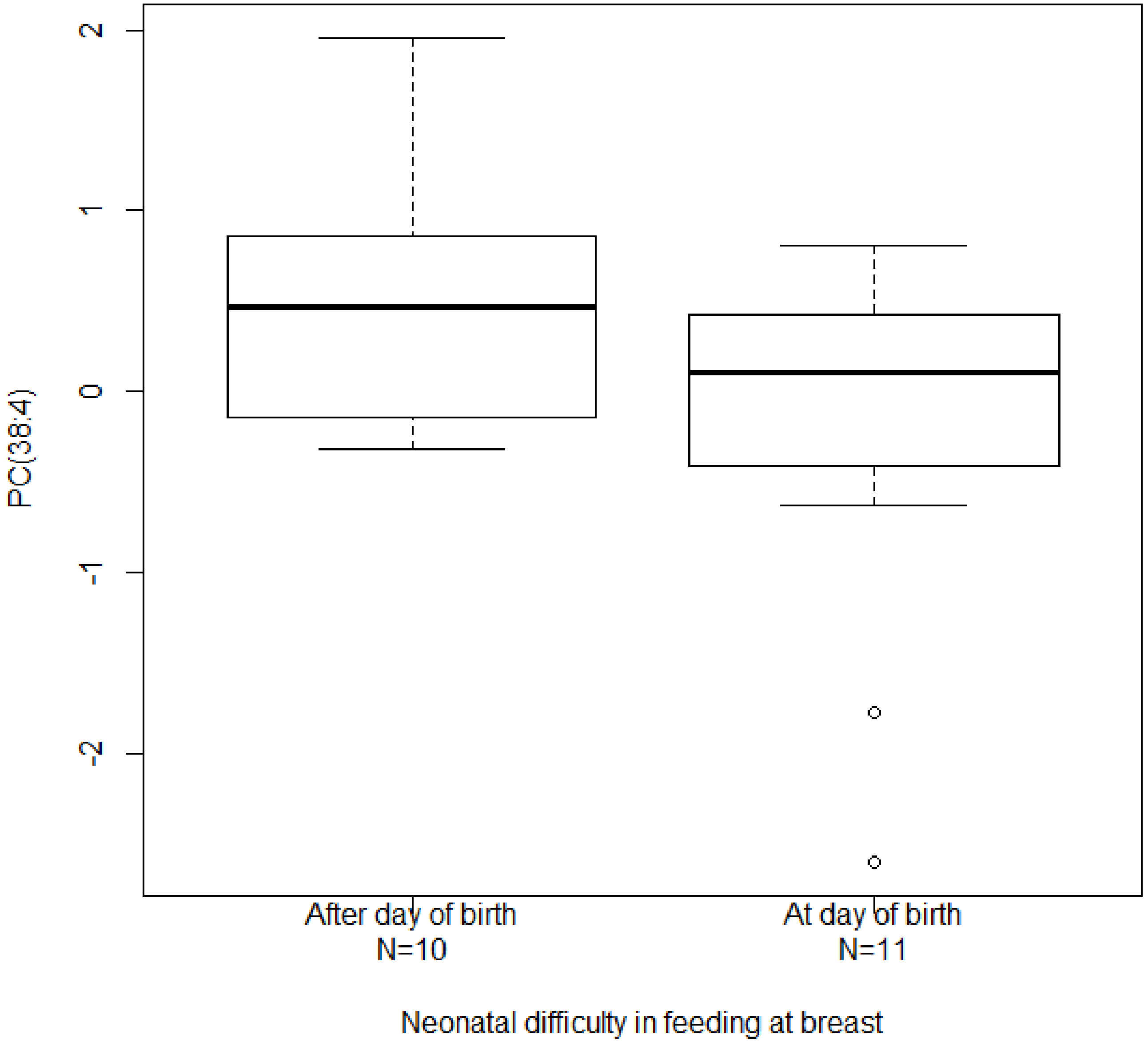

Supplement: Supplementary file 2 — Electronic supplementary material 2 (PDF 492 kb) [file 11306_2020_1763_MOESM2_ESM.pdf]
