# Supplementary material for: Integrating genetics with newborn metabolomics in infantile hypertrophic pyloric stenosis
Source: Metabolomics. 2021 Jan 8;17(1):7. doi: 10.1007/s11306-020-01763-2 (PMC7794101; doi:10.1007/s11306-020-01763-2)
Supplement: Supplementary file 3 — Electronic supplementary material 3 (PDF 252 kb) [file 11306_2020_1763_MOESM3_ESM.pdf]

Metabolites	Beta	SE	P
PC(38:4)	-0.417447983	0.074212694	4.68E-08
PC(36:4)	-0.360019109	0.074829121	2.51E-06
PC-O(36:4)	-0.334980458	0.070485813	3.29E-06
PC(44:1)	-0.332726399	0.076490282	1.94E-05
His	-0.340474498	0.078402335	2.01E-05
PC(38:3)	-0.317460362	0.078842043	7.39E-05
AC(2:0)	-0.251389576	0.067899453	0.000259628
TG(54:5)	-0.30024107	0.082775431	0.000343273
PC(40:6)	-0.244357109	0.067957147	0.000385383
LPC-O(18:1)	0.247402694	0.069332971	0.000426543
PC-O(40:5)	-0.253568131	0.072482487	0.000549785
SM(43:1)	-0.256410325	0.076252749	0.000885216
PC(35:1)	-0.228999476	0.069388771	0.001099162
TG(44:2)	-0.245899186	0.075540733	0.001280257
DG(34:1)	-0.25095923	0.079243678	0.00172016
PC(33:0)	-0.247036566	0.078454189	0.001735096
AC(11:0)	0.243982095	0.077773118	0.001898014
Trp	-0.233347809	0.07521415	0.002127251
Pro	-0.24644616	0.080163593	0.002222615
SM(35:1)	-0.245126055	0.080614851	0.002481112
Creatinine	0.199061107	0.066454321	0.002998625
PC(40:1)	-0.241250959	0.081099934	0.003203659
CE(20:4)	-0.229356199	0.07852112	0.003788526
DG(36:2)	-0.229392421	0.078545798	0.003794794
Met-SO	0.241864062	0.082868464	0.003816618
PC-O(38:5)	-0.235289889	0.08072382	0.003863062
TG(52:2)	-0.231740677	0.079711397	0.003954117
SM(33:1)	-0.222677466	0.07766401	0.004472329
Spermidine	-0.175191418	0.06157235	0.004781585
PC(36:3)	-0.198825204	0.070075598	0.004901453
PC(37:1)	-0.227464857	0.081108258	0.005231597
PC(33:1)	-0.215472682	0.082071826	0.009164899
SM(41:1)	-0.200362514	0.077496627	0.01025914
Tyr	-0.204057306	0.080317558	0.011634002
LPC(16:0)	0.193151508	0.076444418	0.012095095
PC-O(36:5)	-0.179727842	0.071313054	0.012311556
DG(44:3)	-0.183462289	0.076959994	0.01783295
LPC(18:1)	0.168474813	0.072005278	0.020039202
Kynurenine	-0.19365332	0.083667378	0.021406407
DG(36:3)	-0.176863841	0.07785258	0.023908205
SM(43:2)	-0.167952791	0.075172997	0.026299397
TG(52:3)	-0.17867869	0.080962278	0.027761385
Orn	-0.173330547	0.081181298	0.033671397
LPC-O(16:1)	0.154297815	0.07280661	0.034991984
CE(18:2)	-0.143478905	0.068020609	0.035848511
DG(36:4)	-0.161034483	0.077161331	0.037843805
Gln	-0.164348139	0.078858966	0.038116773
TG(50:3)	-0.171722015	0.082877691	0.038766132
PC(33:4)	-0.162149027	0.078653927	0.04022258

TG(48:2)	-0.165898612	0.080886741	0.04125266
LPC(18:0)	0.145283234	0.072740232	0.046812864
H1	-0.16112684	0.082930095	0.053081454
TG(56:6)	-0.139373475	0.073511833	0.059051742
TG(50:2)	-0.152439972	0.080743296	0.060119922
LPC(18:2)	0.12691274	0.068967757	0.06685871
Spermine	-0.133077538	0.077488643	0.087070763
PC-O(34:2)	-0.1233946	0.072457158	0.089735529
SM(30:1)	0.113529094	0.069204093	0.102083211
TG(52:4)	-0.134198326	0.081811275	0.102114748
AC(4:0)	-0.127444708	0.077926911	0.103138978
SM(40:2)	-0.127397068	0.078492353	0.105760622
SM(39:1)	-0.125962176	0.078276484	0.108760076
SM(34:2)	-0.121611731	0.075841417	0.110011076
DG-O(34:1)	-0.123802749	0.077591433	0.111789505
SM(41:2)	-0.124826606	0.079942289	0.119605203
SM(36:1)	-0.121897188	0.079630492	0.126440054
SM(38:2)	-0.121580801	0.081418468	0.135979128
PC(36:2)	-0.083148514	0.058246033	0.154600714
CE(22:5)	-0.106468308	0.075550705	0.159939115
SM(42:2)	-0.105951115	0.075615235	0.162325272
PC(34:3)	0.10124456	0.072929783	0.166225242
TG(55:8)	-0.114579147	0.082860302	0.167885777
SM(32:2)	-0.111451454	0.083514042	0.182625516
PC(39:0)	0.102247081	0.07808041	0.191500115
t4-OH-Pro	-0.091261366	0.07236932	0.208405075
PC(24:0)	0.095815264	0.076221717	0.209836893
AC(18:2)	0.098874028	0.078854389	0.21045742
Asn	-0.097215904	0.078918806	0.21909766
AC(16:0)	-0.101395421	0.08260031	0.220182696
CE(16:1)	-0.097967022	0.083744081	0.243116002
xLeu	-0.091124421	0.078527928	0.246423592
PC(41:3)	0.072216142	0.064913601	0.266930519
Ser	-0.092550271	0.084305532	0.273288951
Cer(42:1)	-0.085564589	0.078388858	0.276021997
AC(18:1)	-0.091177771	0.084614535	0.282206488
ADMA	0.083099063	0.07765939	0.285585209
AC(6:1)	-0.082942375	0.078101274	0.289208481
SM(44:2)	-0.075671132	0.07654795	0.32378386
SM(38:1)	-0.079814916	0.081095054	0.325474988
DG(42:2)	-0.063903544	0.066296151	0.335969511
AC(5:0)	-0.080621959	0.08425743	0.339509678
CE(18:3)	-0.07565655	0.079618171	0.342851115
LPC(16:1)	0.075393777	0.081578656	0.35623083
Cer(43:1)	-0.071765973	0.079328818	0.366461733
AC(7:0)	0.065911446	0.077628623	0.396610745
PC-O(36:2)	-0.062230768	0.077230906	0.421096224
Met	-0.05984496	0.075355268	0.427807103
AC(10:0)	-0.059407243	0.079030978	0.452579495
Cer(42:2)	-0.058787841	0.079080006	0.45789868

PC(32:0)	-0.050252248	0.068374246	0.463013124
Ala	-0.054404184	0.075779847	0.473435235
AC(14:2)	0.051143007	0.072569862	0.481586728
SM(34:1)	-0.048800685	0.073275952	0.505998485
SM(39:2)	0.052150647	0.081009014	0.520282056
CE(22:6)	-0.044746475	0.071253105	0.530546986
LPC(15:0)	-0.046646416	0.075272131	0.535988343
Asp	0.049104795	0.079353194	0.53657015
AC(0:0)	0.051867128	0.085650371	0.54506996
AC(14:0)	0.049599324	0.082439381	0.547922541
AC(5:1)	-0.040035503	0.067499584	0.553605765
LPC(20:1)	0.043304541	0.073919163	0.55848254
Ile	-0.045539533	0.078298385	0.561081862
AC(12:1)	0.040594532	0.071874957	0.572689614
AC(8:1)	-0.047614013	0.08472419	0.574369843
Val	-0.043793084	0.080895806	0.588717588
SM(32:1)	-0.041155186	0.077594578	0.596285974
Lys	-0.039246255	0.079594516	0.622364604
Thr	-0.040780012	0.08373259	0.626447799
PC(32:2)	0.036311743	0.07809554	0.642339642
PC(34:4)	-0.03387436	0.073726121	0.646277714
LPC(14:0)	0.036205464	0.084088993	0.666968587
PC(32:1)	-0.027085567	0.067033216	0.686492965
Taurine	-0.031694824	0.080571401	0.694360254
Gly	-0.03160095	0.080869987	0.696286704
SDMA	0.024684291	0.065578625	0.706913297
PC-O(34:1)	-0.023056763	0.072611003	0.751086321
AC(14:1)	0.020531121	0.072498645	0.777248769
AC(5:0-DC)	-0.020956372	0.08002206	0.793615542
AC(6:0)	0.020634333	0.080977207	0.799061144
PC-O(34:4)	0.019503256	0.080552054	0.808873436
PC(30:0)	-0.017704602	0.077155272	0.818683166
AC(3:0)	-0.016082776	0.070221233	0.819021985
Phe	-0.018472641	0.080978323	0.819645313
Glu	-0.017560965	0.083034625	0.83266949
Histamine	0.017340047	0.08221431	0.833117079
AC(12:0)	-0.012861063	0.06859395	0.851414714
LPC(17:0)	0.013390904	0.077130729	0.862302215
Cer(40:1)	0.012994463	0.079603887	0.870453939
Arg	-0.012632645	0.078498938	0.87227239
PC(29:0)	0.010981267	0.078756713	0.889213736
DG(39:0)	-0.0097841	0.070493772	0.889718373
SM(44:1)	-0.009747451	0.0791129	0.902034138
PC(34:2)	0.006790439	0.062288687	0.913272231
Cit	0.009215488	0.08574267	0.91445153
AC(13:0)	0.008055323	0.085745747	0.925223941
AC(10:2)	0.007013347	0.083678128	0.933268135
Sarcosine	0.001727768	0.081444092	0.98309072
PC-O(32:0)	-0.000789926	0.075199519	0.991626719
