# Supplementary material for: Integrating genetics with newborn metabolomics in infantile hypertrophic pyloric stenosis
Source: Metabolomics. 2021 Jan 8;17(1):7. doi: 10.1007/s11306-020-01763-2 (PMC7794101; doi:10.1007/s11306-020-01763-2)
Supplement: Supplementary file 4 — Electronic supplementary material 4 (PDF 250 kb) [file 11306_2020_1763_MOESM4_ESM.pdf]

Metabolites	Beta	SE	P
PC(38:4)	-0.70689	0.118532	1.28E-08
PC(40:6)	-0.57156	0.095982	4.16E-08
PC-O(36:4)	-0.67959	0.12412	3.44E-07
PC(38:3)	-0.5944	0.123056	5.31E-06
PC-O(40:5)	-0.53648	0.114882	9.93E-06
Creatinine	0.429274	0.093204	1.25E-05
PC(36:4)	-0.58658	0.128462	1.46E-05
His	-0.56837	0.139255	9.25E-05
AC(2:0)	-0.38544	0.097458	0.000149
Spermidine	-0.35846	0.093699	0.000232
PC-O(36:5)	-0.45525	0.124959	0.000434
LPC-O(16:1)	0.435019	0.12084	0.000503
PC(36:3)	-0.41683	0.118534	0.000553
PC(40:1)	-0.40315	0.117744	0.000913
PC(44:1)	-0.43698	0.129671	0.00108
AC(11:0)	0.496643	0.147419	0.00125
PC-O(38:5)	-0.49524	0.152227	0.00157
TG(44:2)	-0.35557	0.111639	0.001952
LPC(16:0)	0.376108	0.11966	0.001955
Trp	-0.41934	0.13646	0.002449
LPC-O(18:1)	0.341193	0.113795	0.003453
PC(35:1)	-0.36759	0.122697	0.003491
DG(34:1)	-0.37827	0.133168	0.005488
TG(50:3)	-0.34397	0.129946	0.009478
Pro	-0.37063	0.141631	0.009628
Tyr	-0.36476	0.142638	0.011374
TG(54:5)	-0.39015	0.151631	0.011626
PC(33:1)	-0.38479	0.152226	0.013172
TG(48:2)	-0.34557	0.137441	0.013596
TG(52:3)	-0.29283	0.118504	0.015214
CE(18:2)	-0.23858	0.096716	0.015395
DG(36:4)	-0.30657	0.125268	0.016199
SM(43:1)	-0.31401	0.129844	0.016588
PC(36:2)	-0.23002	0.094697	0.01699
PC(37:1)	-0.36034	0.152137	0.01892
PC(33:0)	-0.29242	0.123853	0.020241
DG(36:3)	-0.28487	0.12254	0.022258
PC(33:4)	-0.32197	0.140755	0.024347
PC-O(34:2)	-0.28122	0.123264	0.024711
TG(56:6)	-0.27584	0.126708	0.031917
Spermine	-0.28089	0.129035	0.031926
PC-O(36:2)	-0.26916	0.1251	0.033919
AC(12:1)	0.238996	0.111181	0.034088
H1	-0.26	0.122275	0.034835
TG(52:2)	-0.25473	0.120782	0.037517
Gln	-0.27072	0.128471	0.037675
Ala	-0.26321	0.12564	0.038827
Taurine	-0.2894	0.145529	0.049613
DG(44:3)	-0.23567	0.120478	0.05333

Asn	-0.23442	0.123199	0.060031
SM(43:2)	-0.24649	0.129776	0.060561
DG(42:2)	-0.17573	0.094416	0.065807
DG(36:2)	-0.2392	0.128571	0.065852
Orn	-0.26541	0.148264	0.076603
SM(33:1)	-0.24491	0.137772	0.077148
LPC(18:1)	0.214001	0.125689	0.090364
Met	-0.20673	0.12523	0.102019
Met-SO	0.210259	0.129359	0.105831
PC-O(34:1)	-0.19776	0.121524	0.106938
SM(32:2)	-0.24557	0.153793	0.113572
SM(42:2)	-0.20526	0.128924	0.114612
t4-OH-Pro	-0.1959	0.12374	0.116715
CE(22:5)	-0.21155	0.13455	0.119195
CE(20:4)	-0.20201	0.130885	0.12599
AC(16:0)	-0.17232	0.113304	0.131546
TG(50:2)	-0.17514	0.120625	0.148265
xLeu	-0.19837	0.139564	0.156954
SM(44:2)	-0.16424	0.116328	0.161206
AC(5:0)	-0.18243	0.133623	0.173865
TG(55:8)	-0.18221	0.133227	0.174638
TG(52:4)	-0.18134	0.134673	0.181308
PC(34:2)	-0.12769	0.096313	0.188039
AC(8:1)	-0.18324	0.138435	0.188726
LPC(18:0)	0.150641	0.116191	0.197877
PC(24:0)	0.165137	0.130431	0.208514
AC(3:0)	-0.1407	0.111498	0.210064
DG-O(34:1)	-0.16817	0.133991	0.212616
AC(12:0)	0.121599	0.097958	0.217497
Cer(42:2)	-0.16002	0.128915	0.217498
SM(40:2)	-0.15169	0.124044	0.222968
SM(35:1)	-0.15923	0.130746	0.224872
PC(41:3)	-0.14666	0.12216	0.232885
LPC(18:2)	0.158788	0.134028	0.239013
AC(10:0)	0.153175	0.130025	0.2417
SM(38:2)	-0.14642	0.129003	0.257871
SM(41:1)	-0.14156	0.124623	0.25881
LPC(16:1)	0.151288	0.13612	0.267865
CE(16:1)	-0.14401	0.129931	0.269197
Kynurenine	-0.15933	0.145108	0.273652
PC-O(32:0)	-0.12957	0.121535	0.289008
Cer(42:1)	-0.14412	0.137886	0.298522
ADMA	-0.14045	0.135061	0.30109
SM(32:1)	-0.13516	0.131668	0.307202
Phe	-0.14379	0.14087	0.308762
CE(18:3)	-0.13275	0.131612	0.315654
Glu	-0.14464	0.145586	0.321797
AC(14:1)	0.102407	0.103948	0.326991
LPC(20:1)	-0.12482	0.126823	0.327458
Lys	-0.1353	0.138478	0.32984

LPC(17:0)	0.146272	0.150061	0.330993
AC(18:1)	-0.14073	0.145267	0.335094
Asp	-0.12517	0.131336	0.342928
SM(34:2)	-0.11479	0.128517	0.373969
SM(39:2)	0.138355	0.160177	0.389868
SM(41:2)	-0.11939	0.139004	0.391532
PC-O(34:4)	0.109156	0.130268	0.404125
SM(39:1)	-0.11129	0.136125	0.415635
Cer(43:1)	0.096195	0.118195	0.417727
CE(22:6)	-0.08553	0.113382	0.452475
SM(34:1)	-0.08397	0.112676	0.457934
Sarcosine	-0.10162	0.140354	0.469997
PC(29:0)	0.08975	0.12538	0.47583
SM(44:1)	-0.08767	0.127156	0.492175
PC(34:4)	-0.07738	0.113577	0.4973
Ile	-0.09319	0.13809	0.501446
Thr	-0.10224	0.152256	0.503479
Histamine	-0.08481	0.132374	0.522533
SM(30:1)	0.07774	0.125377	0.536686
PC(39:0)	0.067471	0.116485	0.563817
Ser	-0.08892	0.155883	0.569706
PC(32:2)	-0.07848	0.149073	0.599817
PC(32:1)	0.058891	0.113024	0.603527
AC(14:0)	-0.07196	0.144107	0.618675
DG(39:0)	0.054018	0.120338	0.654521
AC(18:2)	-0.05272	0.134146	0.694789
Gly	-0.05003	0.147155	0.734589
AC(6:1)	-0.04089	0.134485	0.761756
AC(6:0)	-0.03642	0.126126	0.773404
LPC(15:0)	-0.03903	0.14337	0.786029
AC(13:0)	0.037171	0.140328	0.791663
AC(0:0)	-0.0359	0.13723	0.794182
AC(7:0)	-0.03562	0.146651	0.808613
Cit	0.03349	0.143678	0.815959
Cer(40:1)	-0.02755	0.131091	0.833979
Arg	0.027724	0.140495	0.843982
LPC(14:0)	0.030324	0.154259	0.844379
AC(14:2)	0.017967	0.100181	0.858049
SM(38:1)	-0.02309	0.131876	0.861196
PC(34:3)	0.017716	0.127014	0.889364
PC(32:0)	0.014773	0.114304	0.89731
AC(5:1)	-0.0161	0.127732	0.899972
SM(36:1)	-0.01567	0.12654	0.901555
Val	-0.01345	0.139504	0.923409
AC(5:0-DC)	0.011847	0.142362	0.933854
AC(4:0)	0.008561	0.119036	0.942813
PC(30:0)	0.005886	0.137623	0.965975
AC(10:2)	-0.00393	0.150518	0.979241
SDMA	0.001795	0.104545	0.98634
