# Supplementary material for: Integrating genetics with newborn metabolomics in infantile hypertrophic pyloric stenosis
Source: Metabolomics. 2021 Jan 8;17(1):7. doi: 10.1007/s11306-020-01763-2 (PMC7794101; doi:10.1007/s11306-020-01763-2)
Supplement: Supplementary file 5 — Electronic supplementary material 5 (PDF 250 kb) [file 11306_2020_1763_MOESM5_ESM.pdf]

Metabolites	Beta	SE	P
PC(44:1)	-0.28248	0.096682	0.00395
SM(35:1)	-0.28817	0.104072	0.005873
PC(38:4)	-0.24929	0.094843	0.009353
SM(43:1)	-0.24832	0.094882	0.009646
TG(54:5)	-0.24112	0.097079	0.013955
PC(36:4)	-0.22747	0.092722	0.015142
SM(41:1)	-0.24524	0.100998	0.016206
PC(41:3)	0.17753	0.074017	0.017816
His	-0.21978	0.094811	0.02165
SM(33:1)	-0.21676	0.094929	0.023616
DG(36:2)	-0.22611	0.101809	0.027647
CE(20:4)	-0.22035	0.100451	0.029588
PC(33:0)	-0.22018	0.102122	0.031553
Met-SO	0.223155	0.106012	0.036727
TG(52:2)	-0.22328	0.106802	0.038033
PC(35:1)	-0.17442	0.084821	0.041257
LPC-O(18:1)	0.182452	0.088953	0.04178
TG(44:2)	-0.20761	0.10135	0.042066
Kynurenine	-0.20294	0.101485	0.047149
ADMA	0.183898	0.096004	0.057055
DG(34:1)	-0.18793	0.100085	0.062096
AC(2:0)	-0.16692	0.089982	0.06417
AC(10:0)	-0.18403	0.100201	0.066824
Cer(43:1)	-0.19125	0.104405	0.068715
AC(4:0)	-0.18414	0.103946	0.078683
AC(18:2)	0.172473	0.097757	0.079538
Pro	-0.16873	0.096137	0.081007
SM(36:1)	-0.17302	0.103267	0.09429
PC-O(36:4)	-0.13476	0.083807	0.109643
AC(7:0)	0.145746	0.090815	0.111443
SM(39:1)	-0.15322	0.097181	0.116745
PC(37:1)	-0.14739	0.095382	0.123834
LPC(20:1)	0.141303	0.092482	0.128346
TG(50:2)	-0.16324	0.108028	0.13389
PC(38:3)	-0.15502	0.102998	0.134106
PC(40:1)	-0.16626	0.110479	0.134186
Asp	0.151654	0.101306	0.135246
Taurine	0.141133	0.095508	0.141335
Orn	-0.14274	0.097178	0.143665
Trp	-0.13064	0.089224	0.144911
SM(30:1)	0.122092	0.084343	0.149556
PC(39:0)	0.149735	0.105375	0.157165
DG(44:3)	-0.14349	0.101698	0.160056
PC(33:1)	-0.1344	0.09623	0.164279
PC(34:3)	0.126003	0.090658	0.166334
LPC(18:1)	0.122382	0.088529	0.168649
DG(36:4)	-0.13024	0.09772	0.184349
SM(41:2)	-0.13137	0.098595	0.184435
Tyr	-0.12684	0.097026	0.192825

DG(36:3)	-0.13099	0.102433	0.202773
TG(52:4)	-0.13193	0.104376	0.207918
SM(43:2)	-0.11803	0.094414	0.213061
LPC(18:2)	0.097718	0.078677	0.216046
LPC(18:0)	0.115756	0.093371	0.216737
SM(34:2)	-0.1158	0.096441	0.231451
TG(52:3)	-0.12758	0.108125	0.238485
Ser	-0.11747	0.10005	0.240756
PC-O(32:0)	0.113149	0.096475	0.242457
SM(40:2)	-0.11723	0.100044	0.242869
PC(32:1)	-0.09279	0.085096	0.277134
AC(12:0)	-0.1019	0.093936	0.279541
CE(18:2)	-0.09767	0.093347	0.296848
AC(6:1)	-0.0987	0.098364	0.317013
AC(5:1)	-0.07865	0.078562	0.31815
AC(0:0)	0.110102	0.110963	0.321325
TG(55:8)	-0.10468	0.105347	0.325492
SM(38:1)	-0.1014	0.103551	0.327479
PC(36:3)	-0.08549	0.08745	0.329751
Spermidine	-0.07997	0.082061	0.331141
PC(32:0)	-0.08246	0.084883	0.332693
AC(16:0)	-0.10605	0.111558	0.341849
PC-O(40:5)	-0.08761	0.093326	0.349176
AC(14:0)	0.09471	0.10154	0.352254
PC-O(34:1)	0.086018	0.092225	0.352294
AC(12:1)	-0.08303	0.094423	0.380623
Val	-0.08786	0.100084	0.381717
Histamine	0.092144	0.106957	0.390125
Gln	-0.08688	0.102351	0.397208
DG-O(34:1)	-0.08183	0.097818	0.404098
SM(38:2)	-0.08513	0.106085	0.423374
LPC(15:0)	-0.06927	0.087786	0.431145
PC-O(38:5)	-0.07276	0.092862	0.434447
TG(48:2)	-0.07864	0.101339	0.438813
PC(32:2)	0.068972	0.090098	0.444979
AC(11:0)	0.067012	0.088533	0.450113
TG(50:3)	-0.08218	0.108942	0.450596
LPC(16:0)	0.066781	0.095879	0.487038
Cer(42:1)	-0.06706	0.096311	0.487196
H1	-0.0767	0.111262	0.491487
PC(33:4)	-0.06553	0.095714	0.494481
CE(16:1)	-0.07424	0.109027	0.4968
TG(56:6)	-0.06078	0.09181	0.508842
LPC(17:0)	-0.05708	0.087008	0.512631
Spermine	-0.06034	0.098956	0.542801
DG(39:0)	-0.0519	0.088728	0.55933
Gly	-0.05448	0.093565	0.560224
PC(34:2)	0.046976	0.081326	0.564254
SM(42:2)	-0.05361	0.095333	0.574576
AC(6:0)	0.058711	0.105283	0.576894

Ala	0.050805	0.095283	0.594536
PC(40:6)	-0.0459	0.090066	0.611012
AC(18:1)	-0.05201	0.103152	0.613914
Cit	-0.0545	0.108613	0.615631
Creatinine	0.045127	0.089651	0.616131
AC(13:0)	0.054906	0.109942	0.61812
t4-OH-Pro	-0.04352	0.088502	0.622831
PC(30:0)	-0.04644	0.094486	0.623666
PC(29:0)	-0.04897	0.10129	0.629259
PC-O(34:2)	-0.04311	0.091094	0.6366
AC(14:2)	0.047767	0.100912	0.636711
AC(5:0-DC)	-0.04586	0.098567	0.642285
PC-O(36:2)	0.042626	0.0998	0.669835
SM(44:2)	-0.04106	0.102501	0.689174
SM(32:2)	-0.03731	0.09779	0.702808
xLeu	-0.03631	0.096223	0.706542
Asn	-0.03891	0.10356	0.707602
AC(3:0)	0.033321	0.092078	0.717872
Glu	0.03477	0.101969	0.733497
AC(5:0)	-0.03689	0.109563	0.736741
PC(24:0)	0.031271	0.094461	0.741015
CE(22:5)	-0.03018	0.092061	0.743403
LPC-O(16:1)	-0.02803	0.090191	0.756316
Sarcosine	0.031043	0.101225	0.759546
Ile	-0.02734	0.096555	0.777251
SM(34:1)	-0.02726	0.097605	0.780341
AC(8:1)	0.027616	0.108128	0.798557
PC-O(36:5)	-0.02188	0.086369	0.800263
Phe	0.024472	0.100346	0.807245
DG(42:2)	-0.02195	0.090686	0.809116
SDMA	0.018549	0.08615	0.829824
SM(32:1)	-0.0206	0.096878	0.83184
LPC(14:0)	0.020296	0.101123	0.840814
CE(18:3)	-0.01906	0.101953	0.851938
AC(14:1)	-0.01796	0.099502	0.857017
SM(39:2)	-0.01643	0.091219	0.85725
Lys	-0.01669	0.096038	0.862257
Arg	-0.01664	0.096215	0.862865
PC(36:2)	-0.01283	0.075171	0.864633
PC(34:4)	-0.01646	0.098554	0.867566
PC-O(34:4)	-0.01645	0.104421	0.874997
Thr	-0.01324	0.100544	0.895214
SM(44:1)	0.011719	0.102962	0.909512
LPC(16:1)	0.010999	0.1019	0.914164
AC(10:2)	-0.00981	0.101649	0.923233
Cer(40:1)	0.009074	0.101415	0.928799
Met	0.008122	0.09451	0.931617
Cer(42:2)	-0.00286	0.102748	0.977804
CE(22:6)	0.000777	0.093703	0.993394
