# Supplementary material for: Integrating genetics with newborn metabolomics in infantile hypertrophic pyloric stenosis
Source: Metabolomics. 2021 Jan 8;17(1):7. doi: 10.1007/s11306-020-01763-2 (PMC7794101; doi:10.1007/s11306-020-01763-2)
Supplement: Supplementary file 6 — Electronic supplementary material 6 (PDF 73 kb) [file 11306_2020_1763_MOESM6_ESM.pdf]

	PC(38:4)			PC(36:4)			PC-O(36:4)			PC(44:1)			Histidine			PC(38:3)			AC(2:0)		
	**BETA**	**SE**	**P**	**BETA**	**SE**	**P**	**BETA**	**SE**	**P**	**BETA**	**SE**	**P**	**BETA**	**SE**	**P**	**BETA**	**SE**	**P**	**BETA**	**SE**	**P**
**Intercept**	-1.20	1.03	2.43E-01	-0.71	1.04	4.97E-01	3.52	1.02	6.18E-04	-6.92	1.05	1.23E-10	-5.21	1.07	1.64E-06	-0.22	1.13	8.49E-01	-3.86	0.94	4.56E-05
**IHPS (case)**	-0.42	0.07	5.55E-08	-0.36	0.07	2.96E-06	-0.34	0.07	3.22E-06	-0.32	0.08	3.33E-05	-0.34	0.08	2.78E-05	-0.32	0.08	8.01E-05	-0.26	0.07	1.72E-04
**YOB (1998)**	-0.32	0.37	3.88E-01	-0.07	0.38	8.62E-01	-0.60	0.39	1.30E-01	-0.19	0.38	6.21E-01	-0.31	0.38	4.13E-01	-0.52	0.43	2.25E-01	-0.10	0.34	7.66E-01
**YOB (1999)**	-0.56	0.35	1.16E-01	-0.47	0.36	1.92E-01	-1.04	0.37	5.56E-03	-0.29	0.36	4.17E-01	0.52	0.36	1.56E-01	0.10	0.40	8.14E-01	-0.23	0.32	4.70E-01
**YOB (2000)**	-0.72	0.40	7.21E-02	-0.60	0.41	1.42E-01	-0.64	0.42	1.30E-01	-1.02	0.41	1.32E-02	-0.60	0.41	1.41E-01	-0.49	0.46	2.85E-01	-0.03	0.36	9.37E-01
**YOB (2001)**	-0.17	0.32	5.98E-01	0.18	0.33	5.73E-01	0.15	0.34	6.55E-01	0.21	0.33	5.19E-01	0.73	0.33	2.65E-02	-0.20	0.37	5.84E-01	0.49	0.29	9.20E-02
**YOB (2002)**	0.41	0.31	1.92E-01	0.52	0.32	1.05E-01	0.54	0.33	1.02E-01	0.30	0.32	3.53E-01	1.26	0.32	1.02E-04	0.49	0.36	1.72E-01	0.10	0.28	7.24E-01
**YOB (2003)**	0.25	0.37	4.95E-01	0.32	0.38	3.97E-01	0.83	0.39	3.48E-02	-0.18	0.38	6.39E-01	0.93	0.38	1.50E-02	0.07	0.43	8.77E-01	-0.05	0.34	8.90E-01
**YOB (2004)**	0.91	0.31	3.63E-03	0.73	0.32	2.23E-02	0.73	0.33	2.76E-02	0.43	0.32	1.75E-01	1.33	0.32	4.16E-05	0.69	0.36	5.51E-02	0.09	0.28	7.44E-01
**YOB (2005)**	0.66	0.33	5.11E-02	0.58	0.34	9.22E-02	0.51	0.35	1.49E-01	-0.30	0.34	3.76E-01	0.85	0.34	1.42E-02	0.39	0.38	3.13E-01	-0.23	0.30	4.41E-01
**YOB (2006)**	0.34	0.29	2.50E-01	0.26	0.30	3.86E-01	0.43	0.31	1.57E-01	0.23	0.30	4.31E-01	1.12	0.30	2.05E-04	0.52	0.33	1.23E-01	-0.02	0.26	9.30E-01
**YOB (2007)**	0.13	0.34	7.00E-01	0.43	0.35	2.17E-01	0.47	0.36	1.94E-01	-0.06	0.35	8.57E-01	1.12	0.35	1.43E-03	-0.05	0.39	9.04E-01	-0.12	0.31	7.05E-01
**YOB (2008)**	0.62	0.30	4.11E-02	0.44	0.31	1.61E-01	0.48	0.32	1.34E-01	0.26	0.31	3.98E-01	1.37	0.31	1.66E-05	0.39	0.35	2.64E-01	0.08	0.27	7.82E-01
**YOB (2009)**	0.92	0.27	7.17E-04	1.03	0.27	2.06E-04	-0.01	0.28	9.78E-01	0.23	0.27	4.06E-01	0.81	0.27	3.37E-03	0.05	0.31	8.68E-01	0.96	0.24	1.00E-04
**YOB (2010)**	0.55	0.26	3.79E-02	0.64	0.27	1.82E-02	-0.39	0.28	1.61E-01	0.32	0.27	2.32E-01	0.80	0.27	3.62E-03	-0.11	0.30	7.15E-01	1.06	0.24	1.39E-05
**YOB (2011)**	1.29	0.28	4.62E-06	1.33	0.28	3.61E-06	0.31	0.29	2.78E-01	0.70	0.28	1.31E-02	1.29	0.28	7.26E-06	0.26	0.32	4.07E-01	1.43	0.25	2.84E-08
**YOB (2012)**	1.13	0.27	4.55E-05	1.19	0.28	2.98E-05	-0.22	0.29	4.49E-01	0.46	0.28	1.00E-01	1.03	0.28	2.92E-04	0.10	0.31	7.39E-01	1.47	0.25	9.60E-09
**YOB (2013)**	0.68	0.28	1.60E-02	0.74	0.29	1.01E-02	-0.51	0.29	8.68E-02	0.28	0.29	3.32E-01	1.08	0.29	2.04E-04	-0.24	0.32	4.50E-01	1.22	0.25	2.39E-06
**YOB (2014)**	0.75	0.27	5.46E-03	0.74	0.27	6.88E-03	-0.59	0.28	3.59E-02	0.18	0.27	5.12E-01	1.01	0.27	2.94E-04	-0.12	0.31	7.00E-01	1.15	0.24	3.36E-06
**sex (male)**	0.06	0.12	5.94E-01	0.07	0.12	5.63E-01	0.34	0.13	6.92E-03	0.30	0.12	1.61E-02	0.15	0.12	2.38E-01	0.22	0.14	1.10E-01	-0.06	0.11	5.67E-01
**c-section**	-0.05	0.10	6.17E-01	-0.08	0.10	4.16E-01	0.04	0.10	6.88E-01	-0.32	0.10	1.97E-03	-0.14	0.10	1.91E-01	-0.02	0.11	8.86E-01	0.20	0.09	2.68E-02
**GA**	0.02	0.02	3.09E-01	0.01	0.02	6.10E-01	-0.08	0.02	1.40E-03	0.18	0.03	3.18E-12	0.11	0.03	2.58E-05	0.01	0.03	7.04E-01	0.08	0.02	2.67E-04
**Parity**	-0.13	0.04	4.60E-03	-0.17	0.05	1.40E-04	-0.14	0.04	1.57E-03	-0.16	0.05	5.04E-04	0.07	0.05	1.27E-01	-0.08	0.05	1.22E-01	-0.04	0.04	3.07E-01
