# Supplementary material for: Integrating genetics with newborn metabolomics in infantile hypertrophic pyloric stenosis
Source: Metabolomics. 2021 Jan 8;17(1):7. doi: 10.1007/s11306-020-01763-2 (PMC7794101; doi:10.1007/s11306-020-01763-2)
Supplement: Supplementary file 7 — Electronic supplementary material 7 (PDF 225 kb) [file 11306_2020_1763_MOESM7_ESM.pdf]

Metabolites	Beta	SE	P
LPC(16:0)	-0.3149	0.1022	0.0022
PC(32:0)	-0.2474	0.0949	0.0094
SM(43:1)	-0.2476	0.1014	0.0149
TG(48:2)	-0.2631	0.1089	0.0161
Met-SO	-0.2541	0.1105	0.0219
LPC(18:0)	-0.2235	0.1019	0.0288
LPC(16:1)	-0.2362	0.1091	0.0309
TG(54:5)	-0.2171	0.1108	0.0506
LPC(17:0)	-0.2054	0.1054	0.0519
LPC-O(16:1)	-0.1948	0.1019	0.0565
SM(36:1)	-0.1998	0.1049	0.0573
Asp	0.2036	0.1082	0.0604
TG(50:3)	-0.2037	0.1092	0.0627
TG(50:2)	-0.1926	0.1067	0.0716
DG(36:2)	-0.1944	0.1084	0.0736
TG(52:3)	-0.1909	0.1067	0.0742
Pro	-0.1885	0.1056	0.0750
PC(36:4)	-0.1805	0.1012	0.0752
PC(30:0)	-0.1882	0.1092	0.0854
PC(44:1)	-0.1739	0.1031	0.0922
PC-O(38:5)	-0.1842	0.1098	0.0942
PC(38:4)	-0.1673	0.0999	0.0948
TG(56:6)	-0.1660	0.1020	0.1044
TG(44:2)	-0.1722	0.1082	0.1123
AC(8:1)	-0.1759	0.1117	0.1160
AC(14:2)	-0.1592	0.1053	0.1314
LPC(18:1)	-0.1528	0.1012	0.1316
AC(5:1)	-0.1596	0.1057	0.1317
DG(39:0)	0.1593	0.1059	0.1330
Kynurenine	-0.1666	0.1125	0.1392
TG(52:2)	-0.1547	0.1058	0.1443
DG(34:1)	-0.1530	0.1071	0.1539
PC(40:1)	0.1577	0.1109	0.1558
PC-O(36:5)	-0.1440	0.1013	0.1558
AC(12:1)	-0.1391	0.0980	0.1564
DG(44:3)	-0.1546	0.1104	0.1623
t4-OH-Pro	-0.1340	0.0958	0.1623
SM(35:1)	-0.1452	0.1064	0.1727
Gln	-0.1414	0.1061	0.1832
CE(16:1)	-0.1453	0.1111	0.1917
Phe	-0.1395	0.1069	0.1924
PC(34:4)	-0.1284	0.1032	0.2141
PC(34:3)	-0.1258	0.1044	0.2289
PC-O(36:4)	-0.1176	0.0998	0.2392
AC(12:0)	-0.1079	0.0955	0.2591
SM(39:2)	0.1222	0.1097	0.2656
Tyr	-0.1180	0.1069	0.2702
PC(35:1)	-0.1098	0.0995	0.2703
DG(36:3)	-0.1212	0.1100	0.2711

Ser	0.1228	0.1117	0.2723
AC(6:0)	-0.1185	0.1083	0.2747
PC-O(40:5)	-0.1113	0.1046	0.2877
SM(43:2)	-0.1133	0.1065	0.2880
LPC(15:0)	-0.1124	0.1072	0.2951
AC(13:0)	-0.1189	0.1151	0.3023
SM(38:1)	-0.1088	0.1071	0.3102
PC(39:0)	0.1123	0.1110	0.3122
DG(36:4)	-0.1111	0.1108	0.3167
SM(33:1)	-0.1053	0.1059	0.3203
PC(36:2)	-0.0838	0.0861	0.3304
PC(32:1)	-0.0901	0.0970	0.3533
TG(55:8)	-0.1008	0.1096	0.3580
SM(32:1)	-0.0988	0.1086	0.3635
SM(40:2)	-0.0926	0.1044	0.3754
SM(41:2)	-0.0934	0.1073	0.3843
SM(38:2)	-0.0909	0.1075	0.3983
Creatinine	-0.0738	0.0880	0.4025
PC(41:3)	-0.0811	0.1009	0.4218
CE(20:4)	-0.0850	0.1058	0.4222
PC(33:1)	-0.0869	0.1125	0.4402
AC(10:0)	-0.0800	0.1044	0.4437
PC(34:2)	-0.0688	0.0904	0.4469
Cer(40:1)	0.0835	0.1112	0.4529
SM(39:1)	-0.0795	0.1073	0.4594
AC(14:1)	-0.0738	0.1011	0.4659
SM(42:2)	-0.0781	0.1080	0.4703
Met	-0.0764	0.1075	0.4773
TG(52:4)	-0.0774	0.1107	0.4846
Spermine	-0.0745	0.1079	0.4904
Gly	-0.0687	0.1069	0.5211
PC(36:3)	-0.0641	0.1009	0.5258
LPC(18:2)	-0.0606	0.0969	0.5324
DG-O(34:1)	0.0659	0.1093	0.5469
AC(7:0)	0.0674	0.1120	0.5474
Sarcosine	-0.0640	0.1084	0.5554
LPC(14:0)	-0.0647	0.1111	0.5606
AC(14:0)	-0.0639	0.1125	0.5702
SM(44:1)	-0.0621	0.1093	0.5704
PC-O(34:2)	-0.0574	0.1060	0.5885
ADMA	0.0581	0.1092	0.5951
Lys	-0.0572	0.1076	0.5953
Histamine	-0.0566	0.1109	0.6100
H1	-0.0552	0.1112	0.6198
SM(34:2)	-0.0530	0.1074	0.6219
Orn	0.0529	0.1087	0.6267
PC(40:6)	-0.0469	0.0971	0.6291
PC(33:4)	-0.0499	0.1112	0.6540
Cer(42:2)	0.0501	0.1118	0.6542
DG(42:2)	-0.0417	0.0960	0.6643

AC(0:0)	-0.0485	0.1132	0.6684
Arg	-0.0476	0.1115	0.6693
Spermidine	0.0370	0.0873	0.6717
Cit	0.0477	0.1133	0.6737
LPC-O(18:1)	-0.0407	0.0976	0.6769
Ala	0.0419	0.1026	0.6830
SM(34:1)	-0.0429	0.1071	0.6885
SM(32:2)	-0.0435	0.1104	0.6938
PC(24:0)	0.0410	0.1102	0.7098
PC-O(34:4)	0.0416	0.1128	0.7126
His	0.0384	0.1044	0.7135
CE(22:5)	-0.0390	0.1066	0.7150
Taurine	0.0393	0.1088	0.7182
AC(4:0)	-0.0372	0.1106	0.7368
Cer(43:1)	0.0369	0.1121	0.7425
AC(11:0)	-0.0341	0.1047	0.7448
AC(3:0)	0.0310	0.0985	0.7530
CE(18:3)	0.0343	0.1096	0.7545
LPC(20:1)	-0.0313	0.1030	0.7612
Glu	-0.0329	0.1110	0.7672
SDMA	0.0266	0.0903	0.7689
PC(33:0)	0.0303	0.1037	0.7700
PC(29:0)	0.0305	0.1075	0.7765
SM(41:1)	-0.0280	0.1065	0.7929
Val	-0.0267	0.1073	0.8039
SM(44:2)	-0.0266	0.1099	0.8087
AC(10:2)	0.0272	0.1151	0.8136
AC(18:1)	0.0249	0.1121	0.8243
xLeu	-0.0216	0.1038	0.8349
Trp	-0.0201	0.0997	0.8401
AC(6:1)	0.0192	0.1126	0.8647
PC(38:3)	-0.0179	0.1103	0.8712
Asn	-0.0162	0.1076	0.8805
CE(18:2)	0.0138	0.0958	0.8855
Ile	-0.0148	0.1035	0.8866
AC(5:0)	-0.0153	0.1139	0.8932
Thr	0.0140	0.1107	0.8994
PC-O(32:0)	-0.0129	0.1070	0.9043
PC-O(36:2)	-0.0132	0.1101	0.9050
AC(2:0)	-0.0088	0.0916	0.9231
Cer(42:1)	-0.0106	0.1104	0.9233
PC(37:1)	-0.0097	0.1072	0.9276
AC(16:0)	-0.0092	0.1092	0.9332
CE(22:6)	-0.0062	0.1006	0.9512
AC(5:0)	0.0050	0.1132	0.9645
PC(32:2)	-0.0036	0.1069	0.9729
SM(30:1)	0.0027	0.1009	0.9788
AC(18:2)	0.0010	0.1042	0.9923
PC-O(34:1)	0.0010	0.1058	0.9924
